# GABA induced by sleep deprivation promotes the proliferation and migration of colon tumors through miR-223-3p endogenous pathway and exosome pathway

**DOI:** 10.1186/s13046-023-02921-9

**Published:** 2023-12-18

**Authors:** Haijun Bao, Zuojie Peng, Xukai Cheng, Chenxing Jian, Xianguo Li, Yongping Shi, Wenzhong Zhu, Yuan Hu, Mi Jiang, Jia Song, Feifei Fang, Jinhuang Chen, Xiaogang Shu

**Affiliations:** 1 Department of Gastrointestinal Surgery, Union Hospital, Tongji Medical College, Huazhong University of Science and Technology, , Jiefang Road No,1277, Hubei, 430022 Wuhan China; 2 Department of Colorectal Surgery, Affiliated Hospital of Putian University, Putian, 351100 Fujian China; 3 Department of Emergency Surgery, Union Hospital, Tongji Medical College, Huazhong University of Science and Technology, Jiefang Road No,1277, Hubei, 430022 Wuhan China

**Keywords:** Sleep deprivation, GABA, Colon cancer cells, Macrophages, Exosomes, miRNA

## Abstract

**Background:**

Research has indicated that long-term sleep deprivation can lead to immune dysfunction and participate in the occurance and progression of tumors. However, the relationship between sleep deprivation and colon cancer remains unclear. This study explored the specific mechanism through which sleep deprivation promotes the proliferation and migration of colon cancer, with a focus on the neurotransmitter GABA.

**Methods:**

Chronic sleep deprivation mice model were used to investigate the effect of sleep disorder on tumors. We detected neurotransmitter levels in the peripheral blood of mice using ELISA. CCK-8 assay, colony formation assay, wound healing assay, and transwell assay were performed to investigate the effect of GABA on colon cancer cells, while immunofluorescence showed the distribution of macrophages in lung metastatic tissues. We isolated exosomes from a GABA-induced culture medium to explore the effects of GABA-induced colon cancer cells on macrophages. Gain- and loss-of-function experiments, luciferase report analysis, immunohistochemistry, and cytokine detection were performed to reveal the crosstalk between colon cancer cells and macrophages.

**Results:**

Sleep deprivation promote peripheral blood GABA level and colon cancer cell proliferation and migration. Immunofluorescence analysis revealed that GABA-induced colon cancer metastasis is associated with enhanced recruitment of macrophages in the lungs. The co-culture results showed that GABA intensified M2 polarization of macrophage induced by colon cancer cells. This effect is due to the activation of the macrophage MAPK pathway by tumor-derived exosomal miR-223-3p. Furthermore, M2-like macrophages promote tumor proliferation and migration by secreting IL-17. We also identified an endogenous miR-223-3p downregulation of the E3 ligase CBLB, which enhances the stability of cMYC protein and augments colon cancer cells proliferation and migration ability. Notably, cMYC acts as a transcription factor and can also regulate the expression of miR-223-3p.

**Conclusion:**

Our results suggest that sleep deprivation can promote the expression of miR-223-3p in colon cancer cells through GABA, leading to downregulation of the E3 ligase CBLB and inhibition of cMYC ubiquitination. Simultaneously, extracellular miR-223-3p promotes M2-like macrophage polarization, which leads to the secretion of IL-17, further enhancing the proliferation and migration of colon cancer cells.

**Supplementary Information:**

The online version contains supplementary material available at 10.1186/s13046-023-02921-9.

## Background

Increasing studies have demonstrated the close relationship between sleep and cancer in recent years.Circadian clocks maintain the homeostasis through temporal regulation of physiology. However, the disruption of sleep rhythms promotes the establishment of cancer features, and the formation of tumors also directly weakens the brain's control of rhythms [1]. Patients with cancer suffer from long-term sleep rhythm disturbance or poor quality sleep, which can be attributed to factors such as pain, the side effects of chemotherapy, and even depression [2]. Sleep deprivation has been reported to account for various malignant tumors, including hematological tumors, lung cancer, and breast cancer [3, 4]. However, the specific relationship between sleep deprivation and the development of colon cancer remains relatively unexplored.

GABA neurons constitute the primary neurons in the wake-sleep circuit [5]. However, the role of GABA in the wake-up loop needs to be identified. Recent studies have shown that sleep deprivation can increase the brain’s discharge frequency of GABAergic neurons [6]. GABA-releasing neurons are instrumental in promoting the transition from non-REM and REM sleep to wakefulness [7]. Zhao et al. reported that the highly activated state of GABAergic neurons in rostromedial tegmental nucleus (RMTg) promotes the transition from rapid eye movement (REM) sleep to awakened and non-REM (NREM) sleep [8]. Another study reported that prolonged waking (sleep deprivation) promotes the expression of GABA_A_ and GABA_B_ in the trigeminal neurons, resulting muscle hypotonia or atonia during sleep [9]. These conclusions indicated that the underlying mechanism by which GABAergic neurons are activated during sleep deprivation. More importantly, apart from the neurotransmitters and secretion functions, GABA regulates tumor proliferation and migration as a signaling molecule in peripheral blood [10]. Tumor cells can synthesize GABA and promote tumor cell proliferation endogenously by themselves [11]. Additionally, Zhang et al. reported that GABA originated from B cells could promote macrophage differentiation while inhibiting the anti-tumor functions of CD8 + T cells [12].

The interaction between primary tumors and local stromal components creates favorable conditions for distant metastasis in the tumor microenvironment (TME) [13]. Exosomes are nanoparticles secreted by cells that facilitate intercellular communication [14]. These exosomes can traverse from donor cells to target cells, potentially promoting tumor proliferation, invasion, metastasis, and drug resistance [15]. Tumor-associated macrophages (TAMs) are the primary component within TME. The infiltration of TAMs in solid tumors has been reported to be associated with poor prognosis [16]. Notably, M2-like macrophages promote cancer’s occurrence and malignant progression by stimulating angiogenesis, increasing tumor cell migration, invasion, and inhibiting anti-tumor immunity [17]. However, the mechanism of intercommunication between macrophages and colon cancer cells remains unclear, and whether GABA in peripheral blood affects this interaction has not yet to be reported.

MiR-223-3p is an inflammation-related microRNA and regulates numerous essential genes involved in inflammation, cell proliferation, and metastasis [18]. MiR-223-3p has been confirmed as a critical inflammasome regulator in macrophages and neutrophils [19]. However, the precise role of miR-223-3p in tumors is still unclear. Studies have reported that miR-223-3p is highly expressed in metastatic gastric cancer [18], whereas another research has suggested that inhibiting the miR-223-3p promoter enhances drug resistance in colon and breast cancer [20]. Notably, miR-223-3p ranks as one of the most abundant microRNAs in extracellular vesicles of peripheral blood and plays a role in intercellular communication [21]. Yang. et al. found that M2-like macrophage-derived exosome miR-223-3p transportation is connected with breast cancer invasion [22]. However, it has been reported that exosomal miR-223-3p from macrophage targets STMN1 and IGF1-R, inhibiting cell proliferation in hepatoma cells [23]. Therefore, the exact function of miR-223-3p in tumor cells and immune cells necessitates further investigation.

The MYC family represents human tumors’ most commonly activated oncoprotein [24]. Studies have shown the role of cMYC in regulating cell proliferation, apoptosis, and chemotherapy resistance in colon cancer [25, 26]. Due to the instability of cMYC protein, strategies aimed at promoting its degradation are considered to be crucial for targeting cMYC as an anti-tumor method. Williams et al. reported that BVES inhibits polyubiquitination of cMYC through PP2A, enhancing cMYC protein stability and thereby inhibiting colitization-induced tumorigenesis [27]. Another study reported that MAGI3, as a novel substrate-binding subunit of E3 ligase, can recognize cMYC and regulate its ubiquitination and degradation, thereby regulating colon cancer progression [28]. Ubiquitination is an essential post-translational modification [29] and has been investigated in the context of cMYC proteins in various ubiquitination mechanisms in multiple cancers [30–32]. However, whether GABA regulates the ubiquitination of cMYC in colon cancer cells has yet to be studied.

In this study, we investigated the effect of sleep deprivation on the increase of GABA in peripheral blood, which promotes the endogenous pathway of miR-223-3p to regulate the proliferation and migration of colon cancer cells. Additionally, miR-223-3p was found to enter macrophages as exosomes, thereby further promoting tumor progression. Mechanistically, GABA promoted the expression of miR-223-3p in colon cancer cells, and miR-223-3p negatively regulated E3 ligase CBLB. Normally, CBLB can bind to cMYC protein and promote its ubiquitination, thereby reducing the stability of cMYC protein and eventually causing proteasomal degradation. GABA can reduce the post-transcriptional modification of cMYC protein by CBLB through miR-223-3p, which maintain the stability of cMYC protein and promote the proliferation and migration of colon tumors. In addition, tumor-derived exosome miR-223-3p activated the MAPK pathway in macrophages, causing M2 polarization. Consequently, macrophages secrete IL-17 and further promote tumor cell proliferation and migration. In conclusion, our study explored the potential mechanism by which sleep deprivation modulates the colon TME through GABA and uncovers a possible strategy for treating colon cancer progression through modification of sleep.

## Methods

### Cell culture

The human colon cancer cells(SW480, LoVo), mouse colon cancer cells(MC38), human monocytic cell line(THP1) and 293 T cells were purchased from American Type Culture Collection (ATCC, USA). All cells were cultured in DMEM/high glucose(Gibco, USA).To induce differentiation into macrophages, THP-1 cells (1 × 10^6^) were treated with 100 ng/mL PMA(MCE,USA) for 24 h. All cells were cultured in a medium supplemented with 10% foetal bovine serum (Gibco, USA) at 37 °C under 5% CO2.

### CRC tissues

The 10 paired of CRC tissues and adjacent noncancerous tissues were collected from Union Hospital, Tongji Medical College, Huazhong University of Science and Technology (Wuhan, China). The consents were obtained from all patients and the use of clinical materials for research purposes were approved by the Ethics Committee of Huazhong University of Science and Technology (Wuhan, China). CRC tissues were taken from the general surgically resected CRC specimens, and the matched adjacent non-cancerous tissues were taken from the site around 5-10 cm away from the edge of the tumors. These specimens were immediately frozen in liquid nitrogen until use. All patients were not received preoperative chemoradiotherapy.

### Experimental animals

Male BALB/C nu/nu mice (4–6 weeks old) and male C57BL/6 mice (4–6 weeks old) were purchased from Vital River Laboratory Animal Technology Co. Ltd. (Beijing, China). The mice were housed in a specific pathogen-free facility under controlled environmental conditions with a room temperature maintained at 23–25℃. All mice have free access to food and water. The care and handling of the mice were processed following the National Research Council’s animal care guidelines and approved by the Institution Animal Care and Use Committee of Tongji Medical College, Huazhong University of Science and Technology.

### Sleep deprivation mouse model

To establish the sleep deprivation mouse model, male C57BL/6 mice (4–6 week old) were randomly divided into an experimental group (*n* = 10) and a control group (*n* = 10). The mice in the experimental group were placed in a sleep deprivation device and maintained under standard environmental conditions of temperature (21 ± 1° C) and relative humidity (50 ± 10%), with a regular 6-h light/2-h dark cycle. The bottom sliding rod rotated while illuminated to hinder mouse sleep. This process lasts for 7 days. The mice in the control group had the same diet as the experimental group, except that they were not subject to daily sleep deprivation.

### AOM/DSS-induced mouse colon cancer model

To investigate the effect of GABA on tumorigenesis, a colon cancer model was generated using AOM/DSS. Male C57BL/6 mice(n = 10) were injected intraperitoneally with AOM (10 mg/kg) (MCE, USA). 1 week after injection, 2.5% DSS(MCE, USA) feeding was added to water for 1 week, followed by normal water feeding for 2 weeks. This DSS feeding pattern was repeated three times. One week after the AOM injected, mice were randomly divided into two groups (*n* = 5), PBS and GABA(50 mg/kg/day). PBS and GABA was intraperitoneally injected twice a week. All mice were sacrificed after 10 weeks and mice colons were harvested to detect polyp development.

### Xenograft subcutaneous implantation model

One of the xenograft subcutaneous implantation model was that, after the sleep deprivation mouse model was established, a total of 1 × 10^6^ colon cancer cells were suspended in 200μL PBS and injected into mice subcutaneously. The tumors volume was measured every 5 days. All mice were sacrificed after 20 days.

The other one was that normal mice were randomly divided into different treatment groups(n = 5). A total of 1 × 10^6^ colon cancer cells, transfected with mimic, siRNA, overexpression plasmids or not, were suspended in 200μL PBS and inject into mice subcutaneously. The tumors volume was measured every week. All mice were sacrificed after 4 weeks.

### Pulmonary metastasis model

One of the pulmonary metastasis model was that, after the sleep deprivation mouse model was established, approximately 5 × 10^5^ colon cancer cells were injected into the tail vein of mice.

The other one was that normal mice were randomly divided into different treatment groups (*n* = 5). Approximately 5 × 10^5^ colon cancer cells, transfected with mimic, siRNA, overexpression plasmids or not, were injected into the tail vein of mice.

All mice were sacrificed after 2 months in order to evaluate pulmonary metastasis. Metastases were fixed with 4% paraformaldehyde for HE staining and IHC.

### Co-culture system and conditioned medium preparation

We used a 0.4 μm pore Transwell chamber (Corning, USA) to build a co-culture system. One of the co-culture systems was that 4 × 10^5^ colon cancer cells (SW480, LoVo) were plated in the bottom chamber of 6-well plates, while 4 × 10^5^ macrophages (THP1) were added into the upper Transwell insert. The other way was that 4 × 10^5^ macrophages (THP1) were plated in the bottom chamber of 6-well plates, while 4 × 10^5^ colon cancer cells (SW480, LoVo) were added into the upper Transwell insert. The cells were co-cultured for 48 h and were used for subsequent experiments.

To collect the conditioned medium (CM) of THP1, SW480 and LoVo cells, 4 × 10^5^ cells were plated in 6-well plates and cultured in 1640 or DMEM/high glucose for 48 h. The supernatant was collected and centrifuged it at 2000 × g for 10 min to eliminate the cells and cell debris. All the CMs were used instantly or frozen at − 80 ℃.

### RNA isolation and qRT-PCR

Total RNA from cultured cells was extracted using Trizol(Japanese TaKaRa) reagent for 5 min at room temperature, then centrifuged at 3000 × g for 15 min at 4℃ to obtain the supernatant. The supernatant was then added to isopropanol and mixed well, then centrifuged at 3000 × g for 10 min at 4℃ to discard the supernatant. The pellet was washed with absolute ethanol and then added to diethylpyrocarbonate in water to measure RNA concentration (ng/μL). The cDNA was then reverse-transcribed by RT Master Mix (TaKaRa, Japan). RT-PCR was performed using SYBR master mix (TaKaRa, Japan) on the Applied Biosystems StepOne-Plus System (American ABI). The expression levels of cellular RNA and mRNA expression were normalized against the housekeeping gene GAPDH. U6 served as a control for miRNA. The primer sequences are listed in Supplementary Table 3.

### Western blotting

Lysing cells and tissues with RIPA buffers containing PMSF(American Sigma) and phosphorylase inhibitors(American Sigma). First, protein specimens were separated by SDS-PAGE. Then, the target protein were transferred to the PVDF membrane (Millipore USA). Then, we incubated the PVDF membrane with the corresponding primary antibody (Table S4) overnight at 4℃. This was followed by incubation with secondary antibody(CST, USA) for 1-h the subsequent day. The protein bands were visualized using ECL (Pierce, USA) and collected by the ChemiDocTm XRS Molecular Imager System (Bio-Rad, USA). Finally, the band densities were analyzed by Image J software. (The details are listed in Supplementary Table 4).

### Transfection assay

HA-tagged CBLB(amino acids 1–982) and its truncations, including CBLB A(amino acids 1–343), CBLB B(amino acids 344–930) and CBLB C(amino acids 931–982),were subcloned into pcDNA 3.1-HA vector. Flag-tagged cMYC(amino acids 1–454) and its truncations, including cMYC A(amino acids 1–368), cMYC B(amino acids 369–421) and cMYC C(amino acids 422–454), were subcloned into pcDNA 3.1-Flag vector.(GeneChem, China). Lipofectamine 3000(Thermo Fisher Scientific, US) was used to transfect plasmids into serum-free Opti-MEM(Gibco).

All the overexpression plasmids targeting cMYC(pcDNA3.1-cMYC) and CBLB (pcDNA3.1-CBLB) were designed and synthesized by GeneChem (China), and the empty plasmid was used as a negative control. MiR-223-3p mimics, miR-223-3p inhibitors and matched control(mimic-NC or inhibitor-NC) were synthesized by RiboBio(Guangzhou, China). All the small interference RNAs were transfected at a final concentration of 50 nM, and the plasmid was transfected at a final concentration of 1.6 μg for 12 well plates. Lipofectamine 3000 transfection reagent (Thermo Fisher Scientific,US) was used for cell transfections according to the manufacturer’s instructions.

A lentiviral vector GV248 containing cMYC shRNA, CBLB shRNA and corresponding negative control were purchased from GeneChem (China). Colon cancer cells were transfected with a lentiviral vector containing cMYC shRNA and CBLB shRNA to establish stable cell lines with downregulated cMYC and CBLB expression. To select stable cell lines, lentiviral-transfected cells were cultured in medium with 1 μg/ml puromycin for 10 days.

Protein and total RNA were extracted after 48 h. (The details are listed in Supplementary Table 5).

### Exosome isolation and treatment

Cells were cultured in medium with exosome-free serum to remove the interference of serum exosomes. Briefly, the serum was centrifuged at 100,000 × g for more than 16 h, and filtered with a 0.22 μm filter (Millipore, USA). The medium was collected after 24–72 h, and the exosomes were isolated according to the instructions on the kit (Invitrogen, California, USA). The medium was centrifuged at 2000 × g for 30 min to eliminate the cells and debris. The total exosome isolation reagent was added to it and incubated between 2 ℃ to 8 ℃ overnight. The exosomes were centrifuged at 10,000 × g for 1 h and resuspended in PBS. qNano and electron microscope were used to quantify the size and concentration of the exosomes and to visualize the morphology of the exosomes. Finally, the exosomes were labeled with PKH26 and were extracted again in the reagent. Exosomes were isolated from 4 × 10^5^ colon cancer cells. THP1 cells were plated on 12-well plates the day before treatment. 100 μg exosomes were added to the plates when THP1 cells were treated by PMA for 24 h. The cells were collected after 48 h for the subsequent experiments.

### MiRNA of exosomes sequencing

After colon cancer cell was treated by GABA, the supernatant of colon cancer cells were collected. Control group was treated by PBS. Then, the exosomes of supernatant were isolated according to the instructions on the kit (Invitrogen, California, USA). Then, we performed miRNA transcriptome profiling by RNA sequencing. Total RNA extraction, RNA sequencing and bioinformatics data analysis were performed by Qijing Biological Technology Co., Ltd(WuHan, China). The small RNA sequencing library adopts the PE150 sequencing scheme and evaluates the quality value of the sequencing library using fastqc. Use bowtie short sequence alignment tool to compare the Rfam library and remove ncRNAs such as rRNA and tRNA. Use miRDeep2 for quantitative analysis of small RNAs, and use DESeq2 for differential expression analysis.

### Bioinformatics analysis

For analysis of public datasets, RNA-seq-based gene expression data in colon cancer were obtained from the GEO database (GSE74602). For gene expression, P < 0.05 was used as the cutoff.

For prediction of miRNA target genes, the overlap of downstream genes of miR-223-3p were predicted by miRDB(http://www.mirdb.org/), miRWalk(http://mirwalk.umm.uni-heidelberg.de/) and TargetScan(https://www.targetscan.org/vert_80/) database. The final prediction results were obtained by intersecting the prediction results of the three databases.

### Coimmunoprecipitation(co-IP)

After transfection, the cells were lysed in RIPA buffer at 4 °C for 1 h. Lysates were centrifuged at 15,000 × g for 15 min at 4 °C, and a sample from the supernatant was collected for further analysis as total lysate. To remove unspecific binding, a prewashing with 0.7 μg/μl IgG-free BSA and 30–50 μl of Protein A/G PLUS agarose was done. Anti-cMYC (6 μg, proteintech) was added to cell lysates and incubated overnight after the addition of Protein A/G PLUS agarose and incubation for 8 h at 4℃. When necessary, a second round of immunoprecipitation was performed. The beads were washed 5 times with 1 ml of immunoprecipitation buffer and then subjected to Western blot analysis.

### CCK-8 assay

Te CCK-8 kit was used to assess cell viability in accordance with the manufacturer’s instructions. In brief, cells (3 × 10^3^ per well) were seeded into 96-well plates (200 μl/well) in culture medium supplemented with 10% FBS with six replicates for each sample. At the appointed time point, 100 μl of fresh medium and 10 μl of CCK-8 solution were added to each well. After incubation for 1 h at 37 ℃, the absorbance was recorded at 450 nm using a Quant ELISA Reader.

### Transwell migration assays

5 × 10^4^ cells in medium without FBS were plated in an 8 μm pore Transwell chamber (BD, USA) for the migration assays. And we placed culture medium supplemented with 10% FBS in the lower chambers. Then, the cells were gently wiped away on the top of the filters after incubation for 24 h for the migration assays. The cells were fixed on the membranes with 5% paraformaldehyde for 20 min followed by staining for 15 min with 0.1% crystal violet. Lastly, five fields of vision were chosen and the number of cells were calculated under the microscope.

### Colony formation assay

SW480 and LoVo cells were cultured in 6-well plates with 500 cells per well and allowed to grow for 14 days in the recommended growth medium. The old medium was replaced with fresh medium every 3 days. Then, the clones were fixed with 4% paraformaldehyde for 20 min and stained with 0.1% crystal violet 15 min. Finally, every wells were chosen and the total number of colonies were counted to evaluate the results.

### Wound healing assays

5 × 10^5^ cells were plated in 6-well plates overnight. 1 mL pipette tips were used to scratch a straight line when the cells achieved 60–80% confluence. Then cells were cultured with medium without FBS. Then, a picture of the cell wound width was taken under the microscope at 0 and 48 h.

### Immunohistochemistry (IHC)

Tissues were formalin-fixed, dehydrated and paraffin-embedded. Then, the tissue sections were incubated with primary antibodies overnight at 4 ℃. Next day, the tissue sections were incubated with HRP-conjugated secondary antibodies for 1 h at 37℃. Then sections were further washed with PBS and distilled water, freshly prepared DAB solution (diaminobenzidine) was subsequently used until the tissue sections were ready to observe. On the one hand, we evaluated the intensity of tissue staining. We calculated the percentage of positive tissue staining (graded as 0, < 5%; 1, 5–25%; 2, 26–50%; 3, 51–75%; and 4, > 75%). SI score was equal to the product of those two. Two experienced pathologists evaluated all the results from the IHC analysis of the tissue sections.(The details are listed in Supplementary Table 4).

### Immunofluorescence (IF)

Colon cancer cells and PMA-pretreated THP1 cells were placed on a glass slide and fixed with 5% paraformaldehyde when the cells reached 60–70% confluence. Blocked the cells with 5% donkey serum for 1 h and incubated with the primary antibody overnight at 4 ℃. The antibodies used for the IF assay were listed in Supplementary Table S 4. The next day, the cells were incubated with the corresponding CY3 secondary antibody (Jackson Immunology Research, Erie, UK) at 37℃for 1 h. After counterstaining the nuclei with DAPI (Sigma, St. Louis, Missouri, USA) for 15 min, the fluorescence images were captured by epifluorescence microscopy (Olympus, Tokyo, Japan).(The details are listed in Supplementary Table 4).

### Chromatin immunoprecipitation (ChIP)

ChIP assay was performed using the SimpleChIP® Plus Enzymatic Chromatin IP Kit (CST, USA). Crosslinked the cells with formaldehyde and sonicate to an average size of 300–500 bp. The lysate was added to the EP tube and incubated with the cMYC antibody. Purified cross-linked DNA released from protein-DNA complexes and further evaluated eluted DNA by qRT-PCR. Used both input and IgG to confirm that the detected signal comes from a specific binding between chromatin and MYC. Used JASPAR to predict the binding site between cMYC and the miR-223-3p promoter. All ChIP assays were repeated 3 times independently.

### Dual luciferase reporter assay for miRNA binding to 3’UTR

To confirm whether CBLB was a target of miR-223-3p,1 × 10^5^ 293 T cells were seeded on 96-well plates and contransfected with dual luciferase reporter plasmid containing CBLB 3’UTR and mutated forms with or without 50 nM miR-223-3p mimic or mimic-NC.After incubation for 48 h,cells were lysed with diluted Passive Lysis Buffer.Luciferase Assay Buffer II was added and used to measure firefly luciferase activity.After stopping the reaction with 1XStop&Glo®Reagent,we measured the Renilla luciferase activity.Activity per well = firefly luciferase activity/Renilla luciferase activity.

### ELISA

After treatment, blood samples were prepared from C57BL/6 mice. Expressions of Gamma-Aminobutyric Acid (GABA), Norepinephrine (NE), Epinephrine (EPI) and Serotonin(5-HT) in blood were assessed using commercially available ELISA kits (Enzyme-linked Biotechnology Co., Ltd., Shanghai).

### Statistical analysis

All data were analyzed by GraphPad Prism5.0 and all assays were repeated at least three times. We used t-test to analyze the frequency of Macrophages in colon cancer tissues and paired adjacent normal tissues. We used the chi-square test to identify the correlation between miR-223-3p and cMYC, CBLB, p-38 and p-ERK in the colon cancer specimens. χ^2^ test was used to analyze the relationship between Macrophages frequency and the clinical features of colon cancer. *P* < 0.05 was considered to be statistically significant and all tests were two-sided.

## Results

### Sleep deprivation promotes occurrence and metastasis of CRC by GABA

**Fig. 1:**
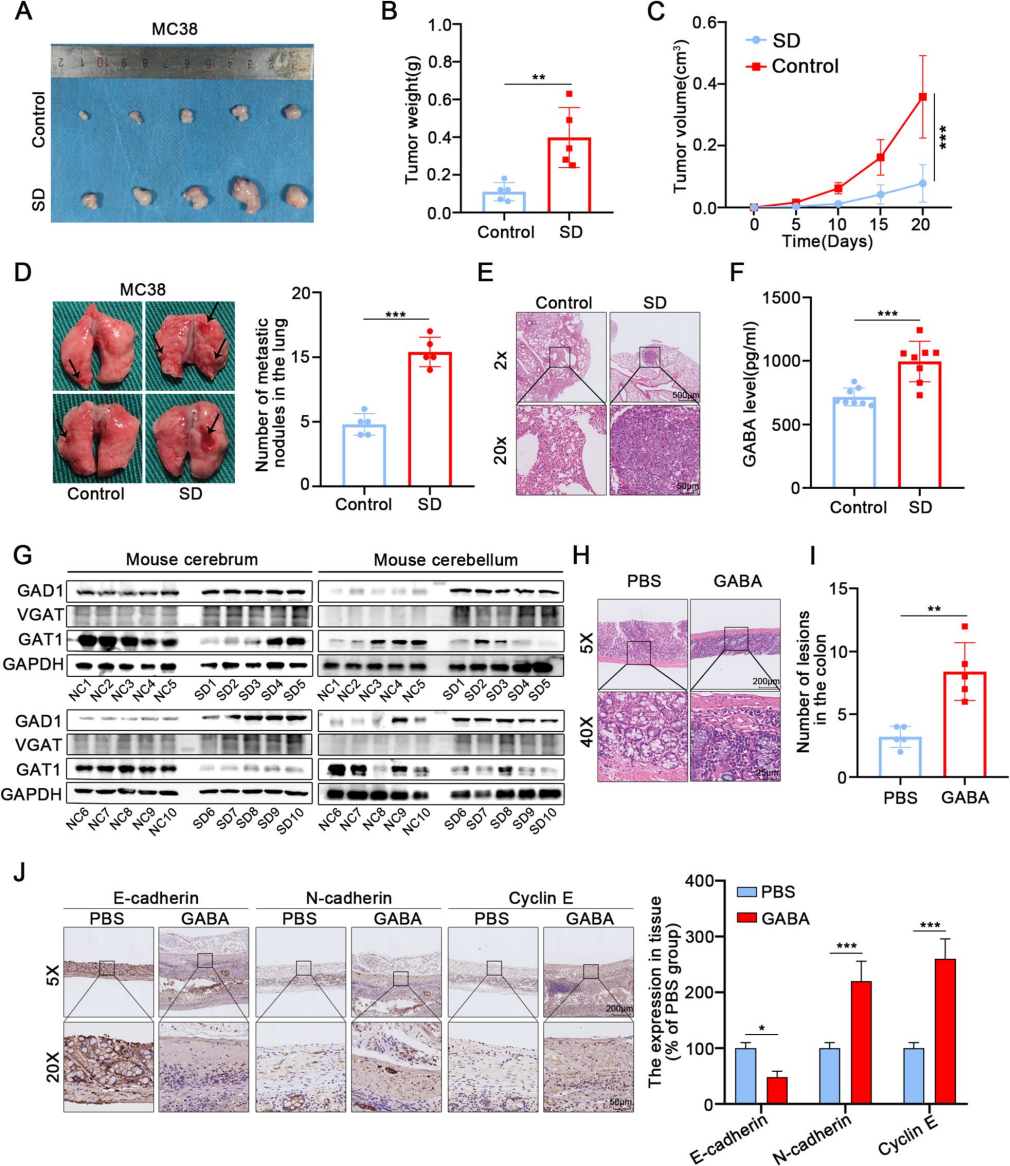
Sleep deprivation promotes occurrence and metastasis of CRC by GABA **A**,** C** Sleep deprivation increased the volume and weight of subcutaneous tumors. **B** The solid tumors were peeled off and weighted. **C** The volume of transplanted tumors was measured by the Vernier caliper every five days. **D** The number of tumors in the lung was counted in control group and SD group. **E** HE staining showed the tumors in the lung of mice. **F** ELISA assay showed the levels of GABA in the serum of SD group and control group mice. **G** The expression of GAD1, VGAT and GAT1 in cerebrum and cerebellum of mice was detected by western blot. **H** HE staining showed the primary tumors from AOM/DSS-induced colonic tumors in PBS group and GABA group. **I** The number of primary tumors in the colon was counted in PBS group and GABA group. **J** The expression level of E-cadherin, N-cadherin and Cyclin E was detected by IHC in primary tumors from respectively intraperitoneal injection PBS or GABA of AOM/DSS model mice. SD:sleep deprivation. All data were revealed as mean ± standard deviation (SD) for no less than three independent experiments. Significant *P* values showed as ^***^P < 0.001.^**^P < 0.01.^*^P < 0.05

We established a chronic sleep deprivation model mouse to explore the effect of sleep disturbance on tumors. The results indicated that in the sleep-deprivated group, volume and weight of subcutaneous colon cancer tumors were increased (Fig. 1A-C). Additionally, more pulmonary nodules and increased infiltration were observed in the sleep-deprivated group compared to the regular sleep group in the metastasis mouse model (Fig. 1D, E).

To further clarify the mechanisms linking sleep disorder and colon tumors, we assessed the levels of several sleep-related neurotransmitters in the serum of sleep deprivation mice, including GABA, NE, EPI, and 5-HT (Fig. 1F, S1A-C). Notably, the serum concentration of GABA significantly increased in the sleep-deprivated group. We examined brain proteins from the control and sleep-deprivated groups to determine the relationship between chronic sleep deprivation and GABA expression. The expressions of glutamate decarboxylase 1 (GAD1), GABA vesicular transporter (VGAT), and GABA transporter 1 (GAT1) in the cerebrum and cerebellum were examined by WB (Fig. 1G). The results showed that elevated expressions of GAD1 and VGAT in the sleep-deprivated group, indicating increased GABA synthesis and transport capacity in the brain tissue. Conversely, GAT1 expression was significantly decreased in the sleep-deprivated group. GAT1 can reuptake GABA in the synaptic space, which suggests that sleep deprivation maintains high GABA levels in the synaptic area, thus promoting GABA entry into the peripheral blood. We also observed the effect of GABA on the AOM-DSS mouse model (Fig. S1D). These results revealed that GABA increased the number of tumor lesions in the colon in mice (Fig. 1H-I, S1E). Furthermore, the immunohistochemical staining results suggested that GABA promoted proliferation and migration in primary colon tumors(Fig. 1J). In conclusion, our findings suggest that sleep deprivation promote colon cancer proliferation and migration by encouraging GABA synthesis and blood entry.

### GABA inhibits ubiquitination of cMYC and promotes proliferation and migration of CRC

Previous studies have identified three kinds of GABA receptors: ionic GABAA, GABAC, and metabolic GABAB receptors. Type A and type B receptors have been widely studied and reported. We screened 30 pairs of colon cancer and paracancerous tissues through the GSE database. The heatmap showed it was the expression of the B receptor but not A receptor in colon cancer tissues that was significantly higher than in the paracancerous tissues (Fig. S2A). Then, we collected seven pairs of colon cancer tissues and detected the expression of GABARAP and GABBR2 in the tissues by qRT-PCR. The results showed that both GABARAP and GABBR2 were highly expressed in the tumor tissues, and the fold change of GABBR2 was more significant (Fig. S2B).

**Fig. 2:**
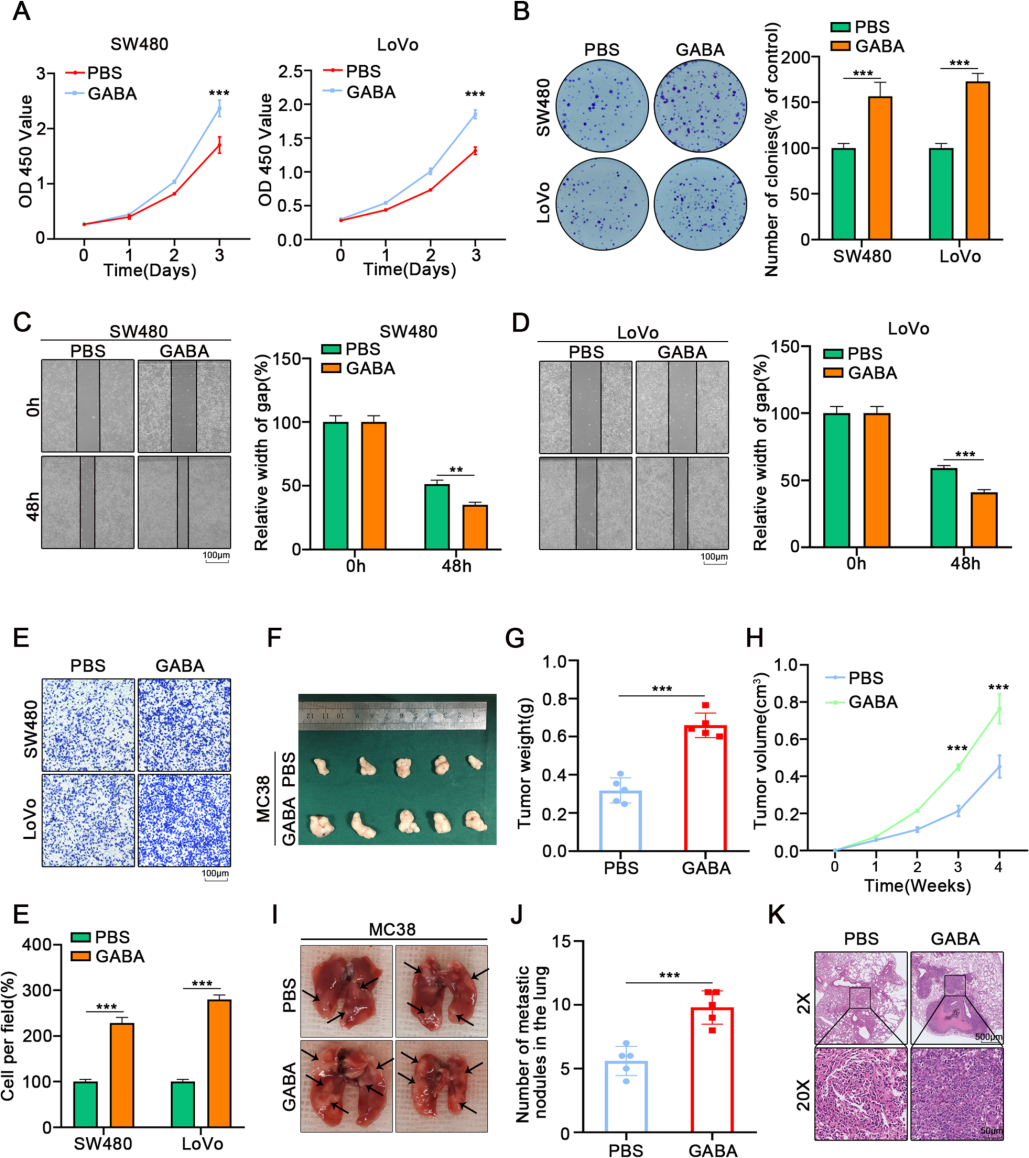
GABA inhibits ubiquitination of cMYC and promotes proliferation and migration of CRC. **A** The proliferation of colon cancer cells was assessed via CCK-8 for 3 days. **B** The proliferation of colon cancer cells was assessed via colony formation assay for 10 days. **C**, **D** The wound healing assays showed that GABA significantly augmented the migrative ability of colon cancer cells. **E** The transwell assays indicated that GABA increased the migrative ability of colon cancer cells. **F**,** H** Treatment with GABA increased the volume and weight of subcutaneous tumors. **G** The solid tumors were peeled off and the weight was measured. **H** The volume of transplanted tumors was measured by the Vernier caliper per week. **I**,** J** The number of tumors in the lung was counted upon treated with GABA or PBS. **K** HE staining showed the tumors in the lung of mice. All data were revealed as mean ± standard deviation (SD) for no less than three independent experiments. Significant *P* values showed as ^***^P < 0.001.^**^P < 0.01

Therefore, we speculated that the B receptor were involved in regulating GABA in colon cancer. To further verify the mechanism of GABA-promoting colon cancer progression, we screened colon cancer cell lines with high expression of B receptors: SW480 and LoVo cells (Fig. S2C). We treated tumor cells with different concentrations of GABA in vitro and selected 100 μM for subsequent experiments (Fig. S2D). The results of CCK-8 assays (Fig. 2A), colony formation assay (Fig. 2B), wound-healing assays (Fig. 2C, D), and transwell assays (Fig. 2E) demonstrated that the proliferation ability and migration ability were enhanced when colon cancer cells were treated with GABA.

The results of Western Blot analysis also showed that GABA treatment upregulated the expression of N-cadherin, vimentin, cyclin D1, and cyclin E while suppressing E-cadherin expression (Fig. S2E). In addition, we found increased volume and weight of subcutaneous xenografts in GABA-treated group compared with the PBS group (Fig. 2F-H). At the same time, the effect of GABA on tumor metastasis was verified by tail vein injection of colon tumor cells (Fig. 2I, H). HE staining indicated that the number and size of pulmonary metastatic nodules in the GABA-treated group were higher than the control group (Fig. 2K). These results confirmed that GABA is able to promote the proliferation and migration of colon tumors.

**Fig. 3:**
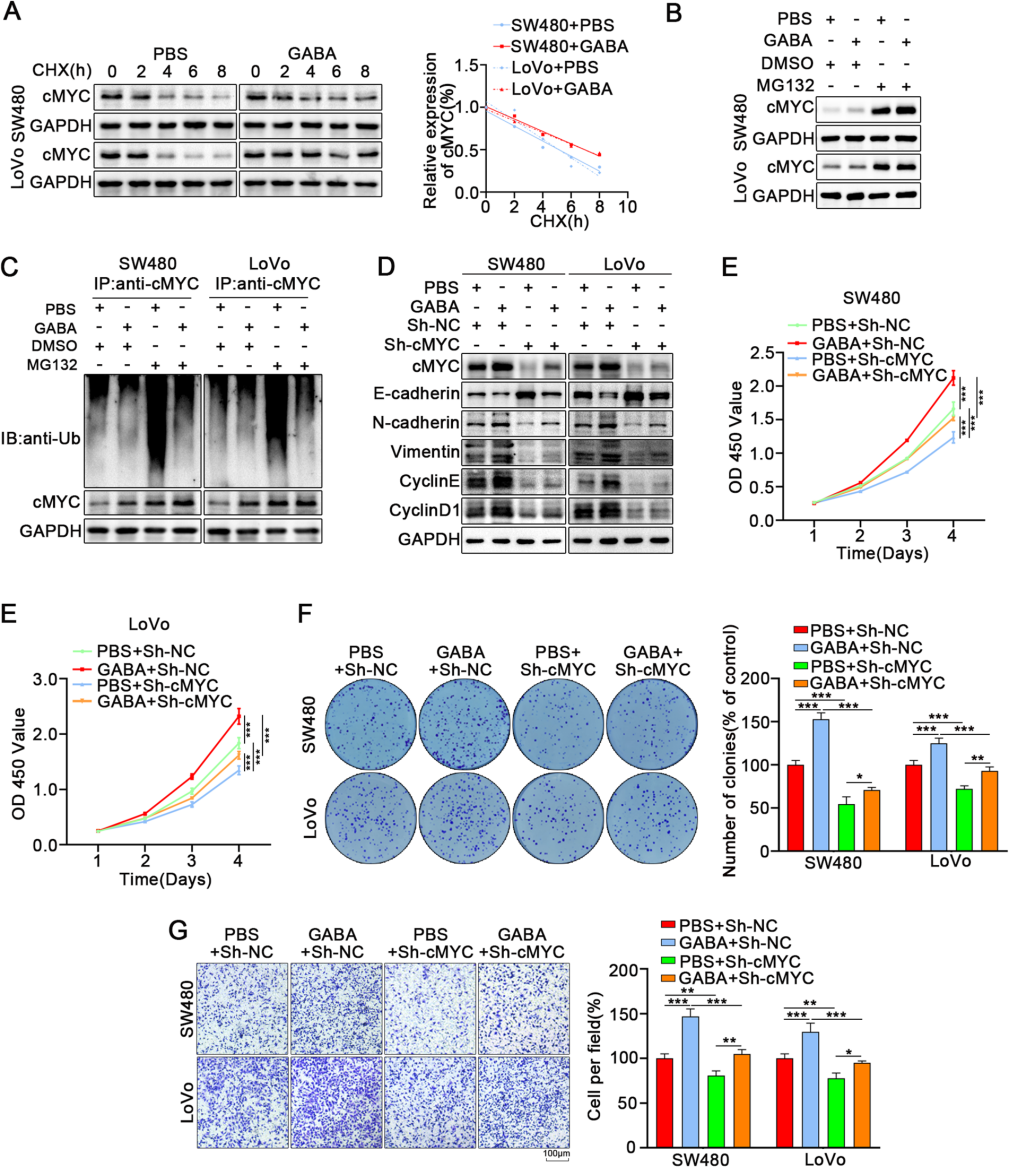
GABA inhibits ubiquitination of c-MYC and promotes proliferation and migration of CRC. **A** Colon cancer cells were treated with GABA followed by treatment with cycloheximide (CHX) for the indicated times. The intensity of cMYC expression at each time point was quantified by densitometry and plotted against time. **B** The expression of cMYC in colon cancer cells treated with GABA for 3 days and then incubated with or without MG132 for 6 h via western blot. **C** Ubiqutin assays of colon cancer cells treated with GABA and MG132. **D** The western blot analysis revealed that cMYC knockdown reversed the effects of GABA-induced promotion of proliferation and migration in colon cancer cells. **E** CCK-8 assays showed that cMYC knockdown reverses the effects of GABA-induced promotion of proliferation in colon cancer cells. **F** Colony formation assays revealed that cMYC knockdown reverses the effects of GABA-induced promotion of proliferation in colon cancer cells. **G** Transwell assays revealed that cMYC knockdown reverses the effects of GABA-induced promotion of migration in colon cancer cells. All data were revealed as mean ± standard deviation (SD) for no less than three independent experiments. Significant *P* values showed as ^***^P < 0.001.^**^P < 0.01.^*^P < 0.05

Studies have suggested that cMYC is abnormally expressed in about 70% of human tumors, regulating cell proliferation, differentiation, metabolism, and apoptosis [33]. We found that GABA treatment can upregulate the expression of cMYC in colon cancer cells. Ubiquitination of cMYC protein is considered as an essential means of targeting cMYC to inhibit tumors and has been widely studied [34]. Therefore, we examined whether GABA inhibited the ubiquitination of cMYC protein. Cycloheximide (CHX), a protein synthesis inhibitor, was used to explore the degradation of cMYC protein. After treatment with CHX, the degradation rate of cMYC protein in GABA group was significantly lower than that in PBS group, indicating that GABA may increase the stability of cMYC protein (Fig. 3A). In addition, MG132, a proteasome inhibitor, take an important role influencing the effect of GABA on the deubiquitination of cMYC protein (Fig. 3B), which affirmed that the inhibition of cMYC degradation by GABA is dependent on protease. We further examined the ubiquitinated form of endogenous cMYC. The cMYC protein was immunoprecipitated with an anti-cMYC antibody, and the ubiquitinated condition of cMYC was detected with an anti-ubiquitinated antibody. In the presence of MG132, the ubiquitinated form of endogenous cMYC in the GABA treatment group was significantly lower than in the PBS group (Fig. 3C). It suggests that GABA treatment reduced the ubiquitination form of cMYC in colon cancer cells and enhanced the stability of cmyc protein. To further explore the effect of GABA treatment on promoting the proliferation and migration of colon cancer cells through regulating cMYC, we knocked down cMYC in GABA-stimulated colon cancer cells. The results revealed that cMYC knockdown weakened the regulation of GABA on the proliferation and migration of colon cancer cells (Fig. 3D-G, S2F).

In summary, our data suggest that GABA enhances the stability of cMYC protein, inhibits its ubiquitination degradation, and promotes colon cancer cell proliferation and migration.

### GABA promotes the recruitment of macrophages by CRC and induces M2 polarization through exosomes

**Fig. 4:**
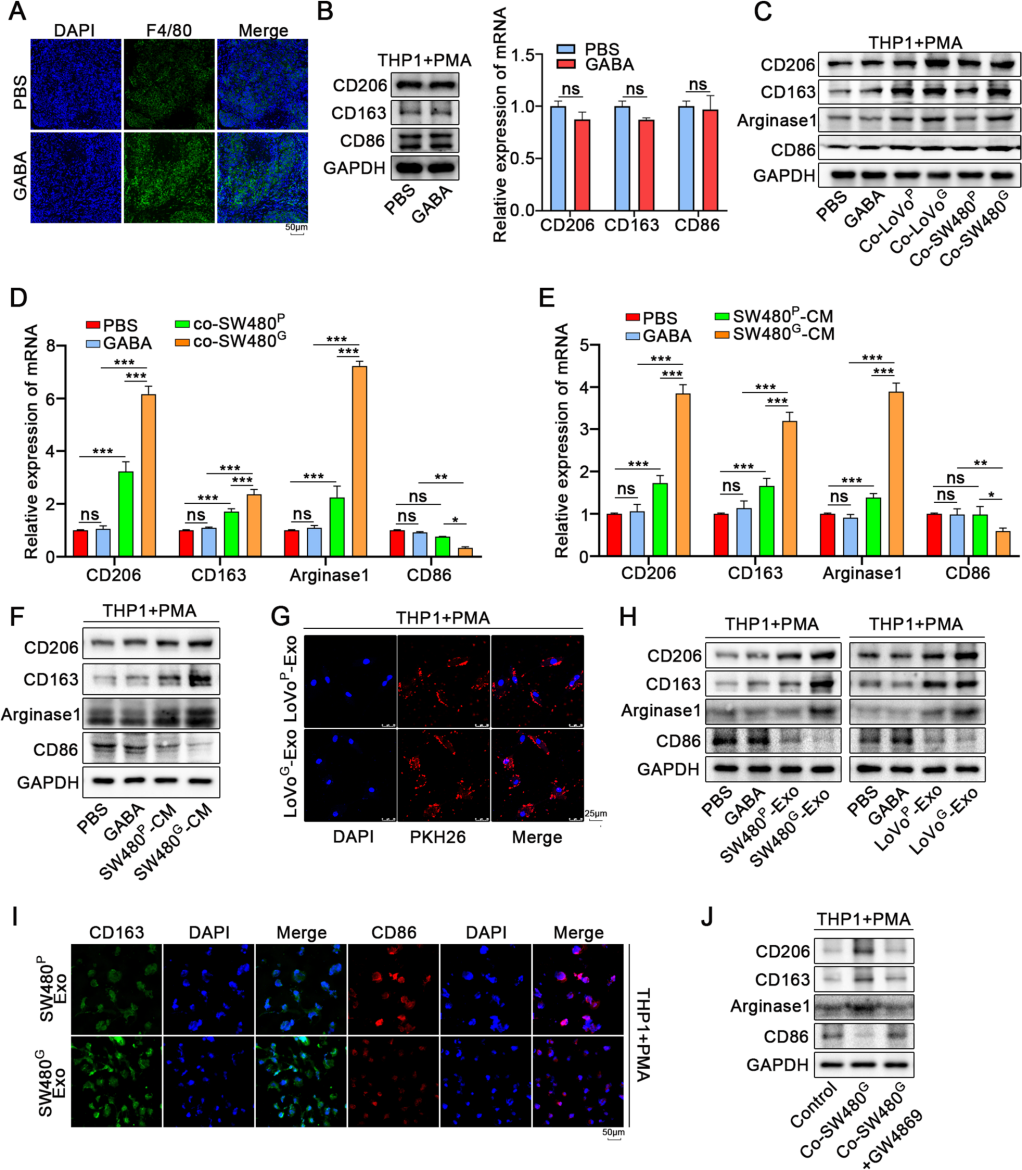
GABA promotes the recruitment of macrophages by CRC and induces M2 polarization through exosomes. **A** Immunofluorescence images showed that macrophages were relatively scarce in lung metastases of PBS group, while were abundant in GABA group. **B** The expression of CD206, CD163, Arginase1 and CD86 of THP1 cells upon administration of GABA were detected by Western blot and qRT-PCR. **C**. The expression of CD206, CD163, Arginase1 and CD86 of THP1 cells co-cultured with GABA-induced colon cancer cells or not by Western blot. **D** The expression of CD206, CD163, Arginase1 and CD86 of THP1 cells co-cultured with GABA-induced SW480 or not was detected by qRT-PCR. **E**, **F** Western blot and qRT-PCR showed that the expression of M2 makers (CD206, CD163, Arginase1) in THP1 cells was increased more significantly when added GABA-induced SW480-CM by qRT-PCR, while M1 maker was decreased. **G** Representative immunofluorescence image showed the internalization of PKH26-labeled LoVo-derived exosomes by THP-1 cells. **H** Western blot showed that the expression of M2 makers in THP1 cells was increased more significantly when added GABA-induced-SW480 derived exosomes, while M1 maker was decreased. **I** Immunofluorescence image showed that THP1 cells exhibited more pronounced M2 polarization when incubated GABA-induced SW480-derived exosomes for 3 days. **J** Western blot showed that GW4869(an inhibitor of exosome secretion) reversed the strengthened M2 polarization of THP1 cells upon incubated GABA-induced SW480-derived exosomes. SW480^P^: PBS-induced SW480 cells. SW480^G^:GABA-induced SW480 cells. LoVo^P^: PBS-induced LoVo. LoVo^G^:GABA-induced LoVo cells. Exo: Exosomes.CM: conditioned medium. All data were revealed as mean ± standard deviation (SD) for no less than three independent experiments. Significant *P* values showed as ^***^P < 0.001.^**^P < 0.01.^*^P < 0.05. ns means the difference was not significant

It has been previously reported that B-cell-derived GABA could affect the classification of macrophages and inhibit anti-tumor immunity, and another study found that GABA secreted by tumor cells were to activate GSK3 β-Pathway to inhibit CD8^+^T cell-mediated tumor immunity [11, 12]. What is mentioned above suggests that GABA is probably involved in the mutual regulation of tumor immunity. We performed immunofluorescence to detect macrophage markers in mouse lung metastases. Interestingly, macrophage infiltration around lung metastases was increased in GABA-treated group mice. (Fig. 4A). Additionally, co-culturing with GABA-induced colon cancer cells increased the migration ability of macrophages (Fig. S4A). It suggests that GABA promotes the recruitment of colon cancer cells to peripheral macrophages, which is related to tumor progression.

TAMs are the primary members of immune cells infiltrating into tumors. M2 macrophages play an irreplaceable role in regulating tumor growth and metastasis. Here, we investigated the polarization of macrophages recruited in the tumor microenvironment. We induced THP1 cells with PMA to form M0 macrophages, and confirmed its morphological changes (Fig. S3B). The augment of M0 marker CD68 was verified by qRT-PCR (Fig. S3C). First, we found that the polarization of THP1 cells treated with GABA alone did not change significantly (Fig. 4B). However, M2 polarization was ensured after co-culturing with normal colon cancer cells through WB and qRT-PCR. Intriguingly, when co-cultured with GABA-induced colon cancer cells, it exhibited more pronounced M2 polarization (Fig. 4C, D, S3D). Combined with previous experimental results, we speculated that GABA could promote the interaction between colon cancer cells and macrophages. We further detected the effect of the conditioned medium of colon cancer cells on macrophage polarization, and the results showed that GABA-treated colon cancer cell medium induced M2 polarization of macrophages more significantly (Fig. 4E, F, S3E, F).

It has been reported that tumor cells can secrete numerous exosomes, which take a great part in cell interaction. We speculated that GABA may promote the secretion of exosomes by colon cancer cells, thereby causing M2 polarization of macrophages. We collected the culture medium of colon cancer cells in the control and GABA treatment groups respectively to extract exosomes, further identified the extracted exosomes (Fig. S 3G- I). Exosomes were labeled with the fluorescent dye PKH26, and our data showed that exosomes derived from colon cancer cells were fused to macrophages (Fig. 4G). The extracted exosomes of colon cancer cells were co-cultured with THP1 cells for 48 h and detected the polarization of macrophages via WB. These results revealed that the M2 polarization of macrophages induced by exosomes secreted by GABA-induced colon cancer cells was more prominent (Fig. 4H). Immunofluorescence showed the same results (Fig. 4I, S3I). In addition, GW4869, an exosome inhibitor, can reverse this M2 polarization effect of tumor cells on macrophages (Fig. 4J, S3J, K).

In conclusion, our data demonstrates that GABA-induced colon cancer cells recruit macrophages in the microenvironment and aggravate the M2 polarization-promoting effect of tumor cells on macrophages through the exosome pathway rather than direct impact of GABA on macrophages.

### Exosome miR-223-3p promotes M2 polarization of macrophages, which aggravates the proliferation and migration of CRC

**Fig. 5:**
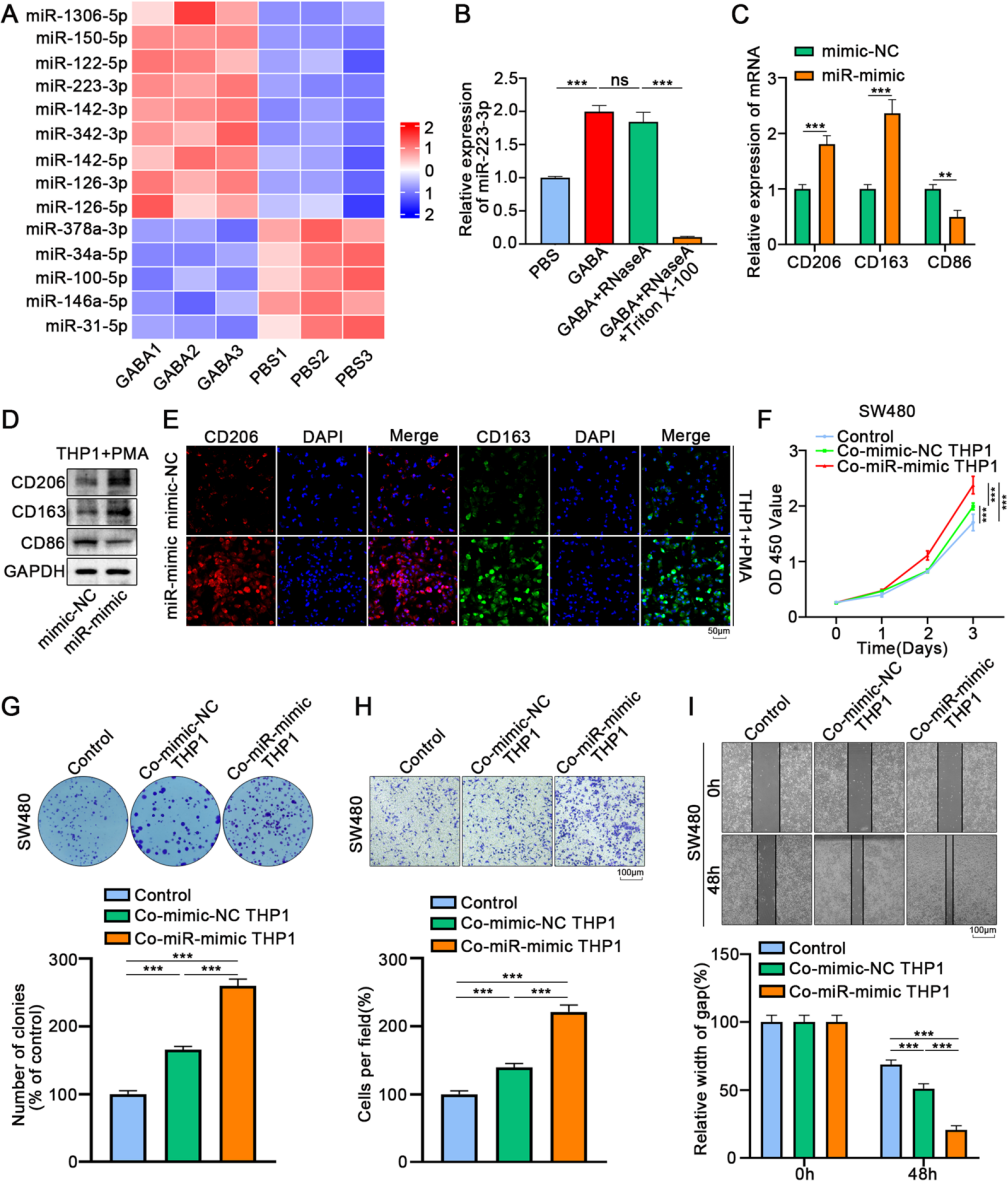
Exosome miR-223-3p promotes M2 polarization of macrophages, which aggravates the proliferation and migration of CRC. **A** A cluster heatmap of the expression profiles of miRNAs in the exosomes derived from PBS-induced LoVo cells and GABA-induced LoVo cells. **B** The qRT-PCR analysis of miR-223-3p in the CM of LoVo cells was treated with RNase A (3U/μg) alone or combined with Triton X-100 (0.1%) for 20 min. **C-D** The expression of CD206, CD163 and CD86 in THP1 cells upon miR-223-3p overexpression or not by qRT-PCR and Western blot. **E** Immunofluorescence assay indicated that miR-223-3p overexpression increased the expression of M2 makers in THP1 cells. **F** The proliferation of SW480 cells co-cultured with THP1 overexpressed miR-223-3p or not was assessed via CCK-8 for 3 days. **G** Proliferation of SW480 cells co-cultured with THP1 cells overexpressed miR-223-3p or not was assessed via colony formation assay for 10 days. **H** The transwell assays indicated that co-cultured with THP1 cells overexpressed miR-223-3p increased the migrative ability of SW480 cells. **I** The wound healing assays revealed that co-cultured with THP1 cells overexpressed miR-223-3p increased the migrative ability of SW480 cells. All data were revealed as mean ± standard deviation (SD) for no less than three independent experiments. Significant *P* values showed as ^***^P < 0.001.^**^P < 0.01.^*^P < 0.05. ns means the difference was not significant

MiRNAs are the most abundant substances in exosomes and participates in regulating various cells. To explore the mechanism of GABA-induced colon cancer cell-derived exosome promotion of M2 polarization in macrophages, miRNA sequencing in exosomes was used to perform large-scale expression profiling(Supplementary Table 1). We screened nine up-regulated miRNAs and five down-regulated miRNAs (Fig. 5A) and verified the sequencing results via qRT-PCR (Fig. S4A). Reports have indicated that mir-150-5p and miR-223-3p are involved in the regulation of macrophage M2 polarization. MiR-150 is an immune-related miRNA, which is upregulated in various cancers [35]. MiR-223 is involved in the regulation of inflammation, tumor proliferation, and invasion after transcription. Zhuang et al. reported that miR-223-3p, a new regulator of macrophages, could inhibit standard M1 polarization and promote M2 polarization [36]. More importantly, miR-223-3p acts as an intermediate communication signal between tumor and immune cells in TME [18]. Therefore, we further detected the content of miR-223-3p and miR-150-5p in exosomes. QRT-PCR results demonstrated that GABA promoted the inclusion of miR-223-3p in exosomes, and the content of miR-223-3p in the medium decreased significantly, while the range of miR-150-5p did not change significantly (Fig. S4B). Additionally, treatment with RNAase A and Triton X-100 significantly reversed the increase of miR-223-3p expression in GABA-induced exosomes, however, RNAase alone had no significant effect (Fig. 5B).

After overexpression of miR-223-3p in macrophages, qRT-PCR, WB, and immunofluorescence results showed that the expression of the M2 polarization marker significantly increased (Fig. 5C-E). We found that miRNAs were correlated with the MAPK pathway via KEGG analysis (Fig. S4C). The activation of the MAPK pathway has been proven to be linkedwith the M2 polarization of macrophages. P38 inhibitor and ERK inhibitor were used in THP1 cells overexpressing miR-223-3p, with the results showing that inhibition of the ERK pathway could effectively reverse the M2 polarization of macrophages induced by miR-223-3p. In contrast, inhibition of the p38 pathway had no significant effect (Fig. S4D). We further co-cultured macrophages overexpressing miR-223-3p with colon cancer cells to observe the feedback effect of macrophages on colon cancer. The results proved that co-cultured with mimic transfected macrophages could significantly improve the proliferation and migration of tumor cells(Fig. 5F-I, S4E-H). Furthermore, the results of subcutaneous tumor implantation in mice indicated that co-culturing with macrophages transfected with mimic could accelerate the growth of colon cancer cells (Fig. S5A-C). The results of lung metastasis of tail vein tumor also showed that co-culturing with mimics transfected macrophages enhanced the migration ability of colon cancer cells (Fig. S5D-F).WB revealed that the expressions of increased N-cadherin, vimentin, cyclin E and cyclin D, decreased E-cadherin, in colon cancer cells co-cultured with THP1 cells transfected with mimic (Fig. S5G).

**Fig. 6:**
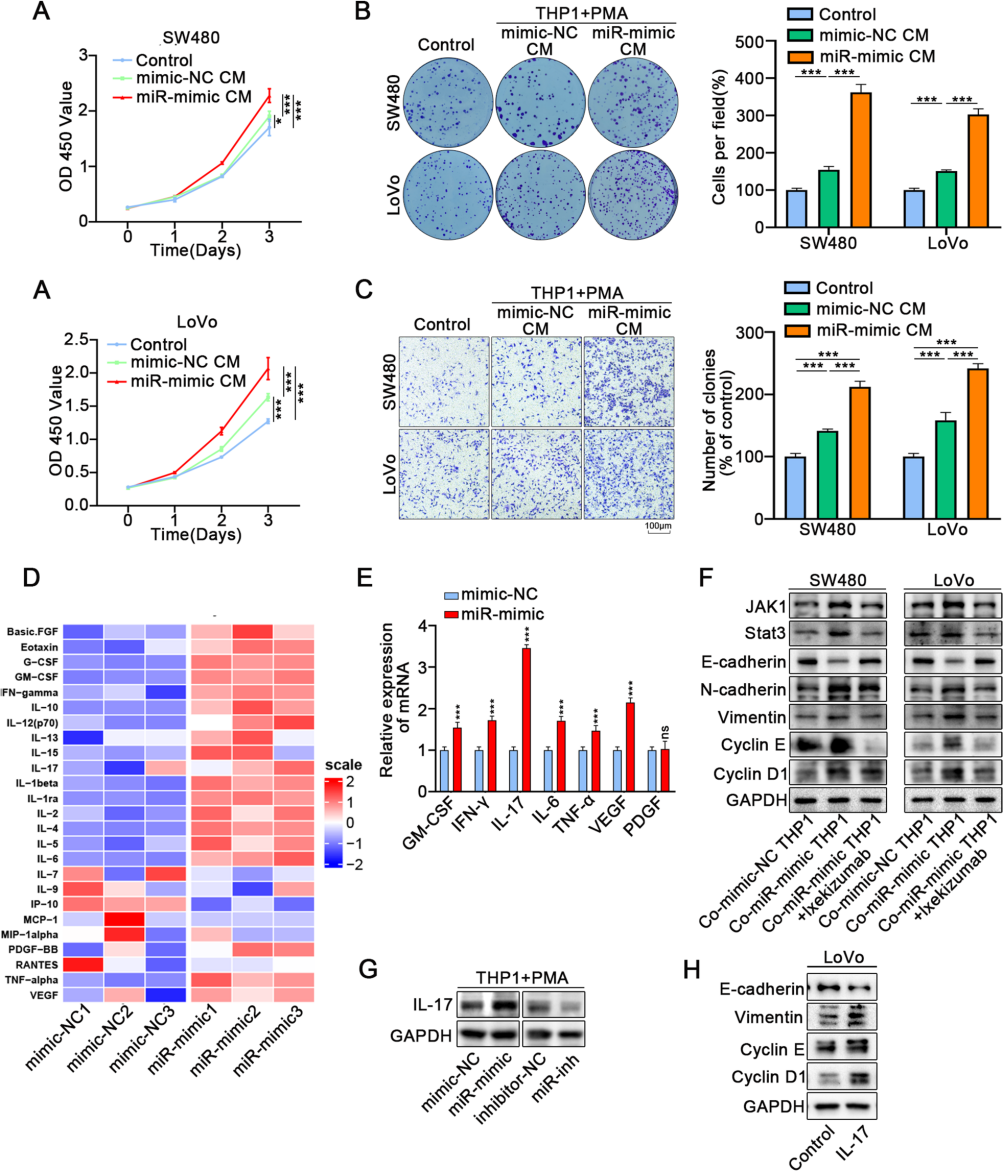
Exosome miR-223-3p promotes M2 polarization of macrophages, which aggravates the proliferation and migration of CRC. **A** CCK-8 assays showed that proliferation of colon cancer cells was significantly augmented when added the CM of THP1 cells overexpressed miR-223-3p. **B** The transwell assays indicated that migrative ability was significantly augmented when added the CM of THP1 cells overexpressed miR-223-3p. **C** The colony formation assay indicated that migrative ability was significantly augmented when added the CM of THP1 cells overexpressed miR-223-3p. **D** A cluster heatmap of the expression profiles of cytokines in the CM of THP1 cells upon overexpression miR-223-3p or not via ELISA assays. **E** The expression of cytokines in THP1 cells upon overexpression miR-223-3p or not was detected by qRT-PCR. **F** Western blot indicated that inhibition of IL17 blocked the altered expression of E-cadherin, N-cadherin, Vimentin, Cyclin E and Cyclin D1 in colon cancer cells co-cultured with THP1 cells overexpressed miR-223-3p. **G** Western blot showed that the expression of IL17 was conspicuously increased or decreased in THP1 cells upon miR-223-3p overexpression or knockdown in THP1 cells, respectively. **H** The expression of E-cadherin, Vimentin, Cyclin E and Cyclin D1 in colon cancer cells upon administration of IL17 were detected by Western blot. All data were revealed as mean ± standard deviation (SD) for no less than three independent experiments. Significant *P* values showed as ^***^P < 0.001.^**^P < 0.01.^*^P < 0.05. ns means the difference was not significant

We also collected mimic transfected macrophage-conditioned medium for culturing colon cancer cells. Results showed that the medium of macrophages transfected with mimics significantly enhanced the proliferation and migration of colon cancer cells (Fig. 6A-C, S5H). WB also revealed the same results (Fig. S 5I). We further measured the level of cytokines in a macrophage culture medium with a cytokine Chip (Fig. 6D). At the same time, qRT-PCR was performed to verify the mRNA level of cytokines, and we found that IL-17 increased most significantly (Fig. 6E). The expression of IL-17 increased or decreased upon miR-223-3p overexpression or inhibition in macrophages (Fig. 6G). IL-17 is a typical pro-inflammatory cytokine, which plays a regulatory role in host defense, tissue repair, inflammatory immunity and tumor progression [37]. Moreover, IL-17 augmented the expression of vimentin, CyclinD1, and cyclinE, while attenuating the expression of E-cadherin in colon cancer cells (Fig. 6H). IL-17 was reported to activate JAK-STAT3 and NF- κ B, and other signaling pathways [38]. It can also promote the expression of PD-L1 and cause tumor immune escape [39]. Isibizumab was used to block the IL-17 receptor in colon cancer cells. The results of WB analysis suggested that the JAK-STAT3 pathway were inhibited, and the expression of the marker proteins of proliferation and migration were also reversed (Fig. 6F).

In conclusion, our data suggested that tumor-derived exosomes miR-223-3p activates the MAPK pathway of macrophages to cause M2 polarization and secrete IL-17 to aggravate the proliferation and migration of colon cancer.

### GABA promotes the proliferation and migration of CRC by inhibiting ubiquitination of cMYC through miR-223-3p

**Fig. 7:**
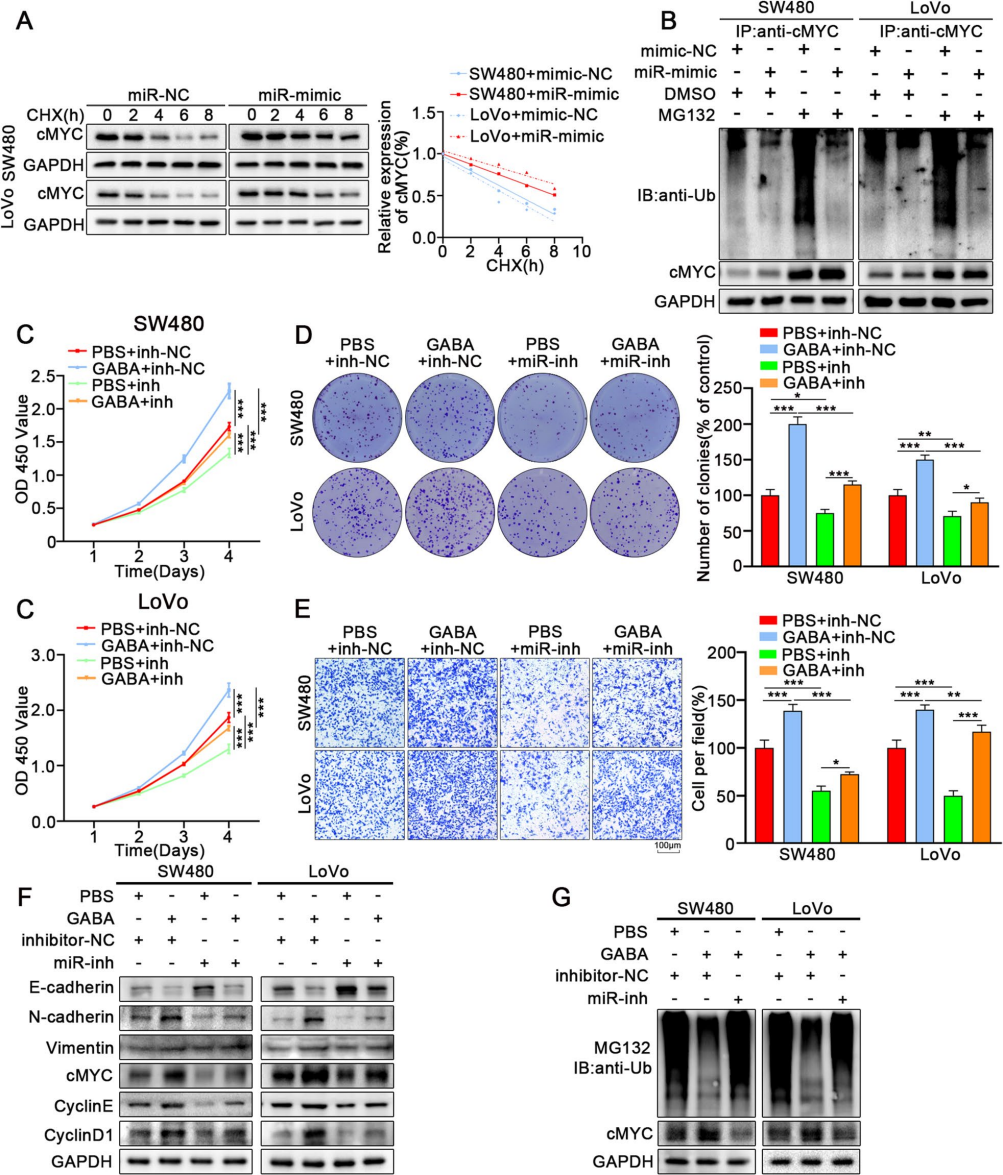
GABA promotes the proliferation and migration of CRC by inhibiting ubiquitination of cMYC through miR-223-3p. **A** Colon cancer cells were overexpressed miR-223-3p followed by treatment with cycloheximide (CHX) for the indicated times. The intensity of cMYC expression at each time point was quantified by densitometry and plotted against time. **B** Ubiqutin assays of colon cancer cells transfected with miR-mimic followed by treatment with MG132. **C**. CCK-8 assays revealed that knockdown miR-223-3p reversed the GABA-induced promotion of proliferation in colon cancer cells. **D** The colony formation assay indicated that knockdown miR-223-3p reversed the GABA-induced promotion of proliferation in colon cancer cells. **E** The transwell assays showed that knockdown miR-223-3p reversed the GABA-induced strengthened migrative ability of colon cancer cells. **F** Western blot showed that inhibition of miR-223-3p reversed the GABA-induced promotion of proliferation and migration in colon cancer cells. **G** Ubiqutin assays indicated that inhibition of miR-223-3p blocked the altered ubiquitination of cMYC in colon cancer induced by GABA. All data were revealed as mean ± standard deviation (SD) for no less than three independent experiments. Significant *P* values showed as ^***^P < 0.001.^**^P < 0.01.^*^P < 0.05

Although miR-223-3p is a conservative anti-inflammatory miRNA, studies have indicated its function in tumor proliferation and invasion in prostate cancer, breast cancer, and other tumors [22, 40]. Therefore, we further explored whether the endogenous expression of miR-223-3p in colon cancer cells impacted on tumor proliferation and migration. Results showed that overexpression of miR-223-3p can promote the proliferation of colon cancer cells (Fig. S6A, B), while increasing the migration ability of colon cancer cells (Fig. S6C, D). Our previous data proved that GABA could increase the stability of cMYC protein in tumor cells and reduce ubiquitination degradation. Notably, after treating with CHX in miR-mimics transfected colon cancer cells, the degradation rate of cMYC was also significantly slower than that of the control group (Fig. 7A). Immunoprecipitation also showed that the overexpression of miR-223-3p could reduce the ubiquitinated form of cMYC in tumor cells (Fig. 7B). This suggests that overexpression of miR-223-3p inhibits the normal ubiquitination and degradation process of cMYC protein, resulting in the relative increase of cMYC protein expression in tumor cells.

In this study, we intended to investigate whether GABA promoted the proliferation and migration of colon cancer by regulating the ubiquitination of cMYC protein through miR-223-3p. Inhibition of miR-223-3p can significantly reverse the promotion of GABA on the increase and migration of SW480 and LoVo cells (Fig. 7C-F). GABA inhibited the ubiquitination and degradation of cMYC protein while inhibition of miR-223-3p reversed the inhibitory effect of GABA on cMYC ubiquitination (Fig. 7G).

In conclusion, our data suggest that GABA promotes the proliferation and migration of colon cancer cells by stimulating the endogenous expression of miR-223-3p and the stability of cMYC proteins.

### CMYC can reverse regulate the expression of miR-223-3p

Coincidently, we found that the expression of miR-223-3p increased or decreased upon cMYC overexpression or inhibition in colon cancer cells (Fig. S6E, F). As a transcription factor, cMYC is involved in the regulation of a variety of tumors. Therefore, cMYC may acts as a transcription factor to regulate the transcription of miR-223-3p.

Data from the Jaspar database indicated a potential binding site for cMYC in the miR-223-3p promoter (Fig. S6G). Chip assays showed that compared with IgG-bound samples, cMYC-bound complexes were significantly enriched in the promoter region of miR-223-3p (Fig. S6H).

In conclusion, these data suggest that cMYC regulates miR-223-3p reversely.

### Mir-223-3p targets E3 ligase CBLB to regulate ubiquitination of cMYC

**Fig. 8:**
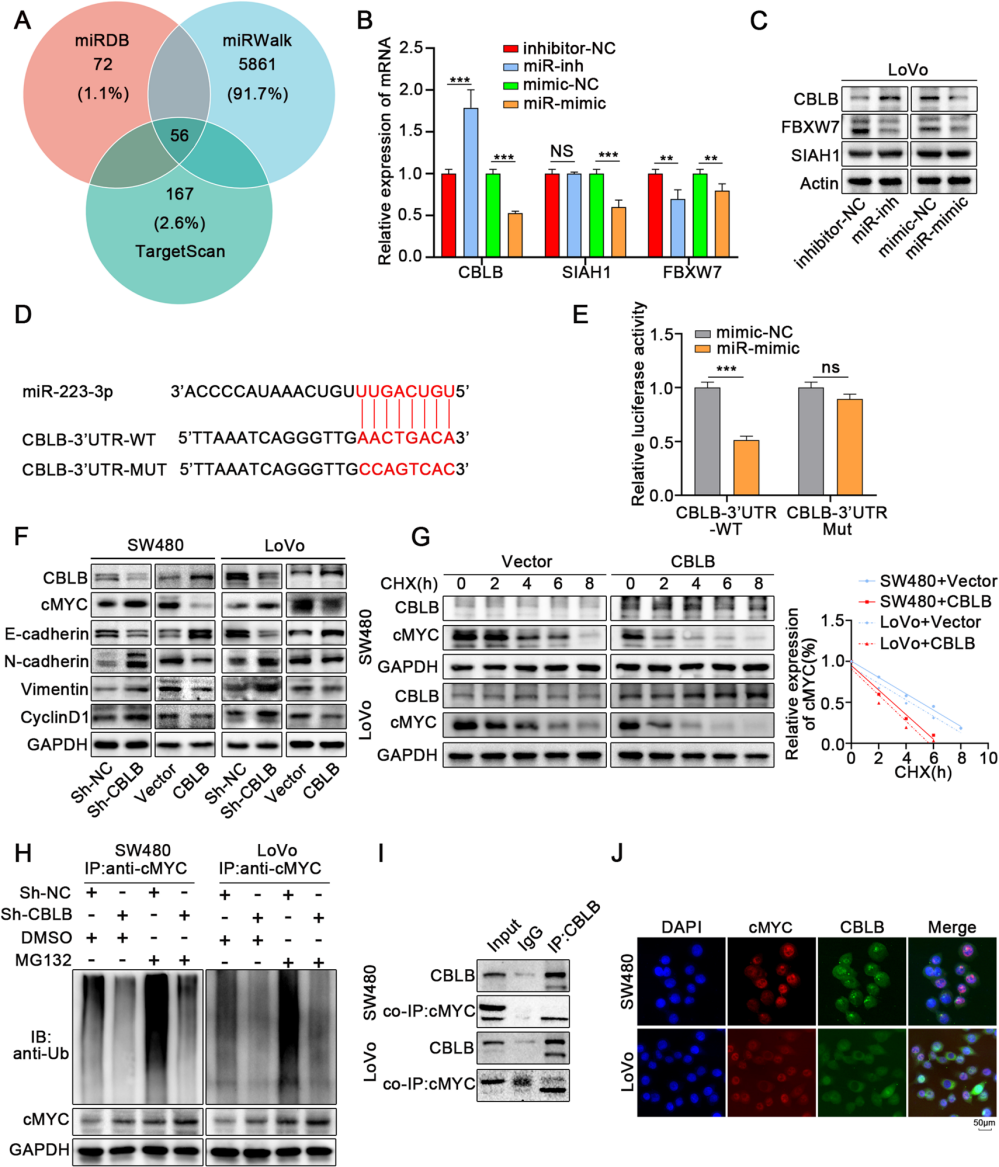
Mir-223-3p targets E3 ligase CBLB to regulate ubiquitination of cMYC. **A** Venn diagram indicated that the overlap of downstream genes of miR-223-3p predicted by miRDB, miRWalk and TargetScan database. **B-C** qRT-PCR and western blot showed that the expression of CBLB was conspicuously decreased or increased upon miR-223-3p overexpression or knockdown respectively in LoVo cells, while the difference of SIAH1 and FBXW7 was not significant. **D** A miR-223-3p binding site on the 3'UTR of the CBLB mRNA was predicted by TargetScan database. **E** The binding of miR-223-3p on CBLB 3’UTR was evaluated by dual luciferase reporter assay.293 T cells were transfected with reporter plasmid containing wild type CBLB 3'UTR(WT) or mutant type (MUT) respectively, followed by transfection with mir-223-3p mimic (miR-mimic) or negative control (miR-NC). **F** Western blot showed that the expression of proliferation and migration markers and cMYC was significantly decreased or increased in colon cancer cells upon CBLB overexpression or knockdown in colon cancer cells. **G** Colon cancer cells were overexpressed CBLB followed by treatment with cycloheximide (CHX) for the indicated times. The intensity of cMYC expression at each time point was quantified by densitometry and plotted against time. **H** Ubiqutin assays of colon cancer cells transfected Sh-CBLB followed by treatment with MG132. **I** CO-IP and Western blot showed that endogenous cMYC and CBLB bind to each other. **J** Immunofluorescence images showed colocalization of CBLB and cMYC in colon cancer cells. All data were revealed as mean ± standard deviation (SD) for no less than three independent experiments. Significant *P* values showed as ^***^P < 0.001.^**^P < 0.01. ns means the difference was not significant

To further clarify the specific mechanism of miR-223-3p regulating cMYC ubiquitination, we screened the target genes of miRNA from miRDB, miRWalk and TargetScan databases respectively (Fig. 8A). Notably, there are 56 downstream target genes in the intersection, three of which related to ubiquitination regulation, namely FBXW7, CBLB and SIAH1. QRT-PCR and WB results demonstrated that CBLB differentiates under the intervention of mimic and inhibitor, while both FBXW7 and SIAH1 performed poorly (Fig. 8B, C). Together, we also found that miR-223-3p may bind onto the 3'UTR region of the mRNA of CBLB (Fig. 8D). To confirm that CBLB was a target of miR-223-3p, 293 T cells were transfected with dual luciferase reporter plasmid containing wild-type CBLB 3'UTR (WT) or mutated type (MUT), followed by transfection with miR-223-3p mimic (miR-mimic) or negative control (mimic-NC). The results showed that the luciferase activity in 293 T cells was reduced upon the overexpression of miR-223-3p in the WT group but not in the MUT group (Fig. 8E).

Following, we verified the function of CBLB in colon cancer. CBLB is a proto-oncogene considered as a new target for tumor immunotherapy [41]. However, its expression in colon cancer has not yet been reported. We collected colon tissue samples from 10 patients with colon cancer. WB analysis results showed CBLB was highly expressed in the non-cancerous tissue of colon but lower in the tumor tissues (Fig. S7A). Immunohistochemistry also showed the same results (Fig. S 7B). We also detected the expression of CBLB in subcutaneous colon cancer tumors of control group and SD group. WB analysis results showed that sleep-deprivation decreased the expression of CBLB in subcutaneous colon cancer tumors, while cMYC was highly expressed in subcutaneous colon cancer tumors of SD group (Fig. S 7C). We further transfected SW480 and LoVo cells with CBLB overexpression plasmid and knockdown lentivirus. We found that knockdown of CBLB enhanced the proliferation and migration of tumor cells, while overexpression had the opposite effect (Fig. S7D-F, S8A-C). WB analysis results revealed that knockdown of CBLB increased the expression of cMYC and promoted the expression of proliferation and migration marker proteins, while overexpression of CBLB led to the opposite effect (Fig. 8F). Immunofluorescence results also indicated that downregulating the expression of CBLB promoted the proliferation and migration of colon cancer cells (Fig. S9A). Whereas, the results of subcutaneous tumor formation in mice also revealed that the expression of CBLB was negatively correlated with tumor proliferation (Fig. S9B-D).

We further explored the specific mechanism of cMYC down-regulation induced by CBLB with CHX to detect the degradation of cMYC protein. It was found that the degradation rate of the cMYC protein was significantly faster when CBLB was overexpressed in colon cancer cells (Fig. 8G), compared to the cMYC protein, which was considerably slower when CBLB was downregulated (Fig. S9E). This indicated that CBLB reduced the stability of the cMYC protein. In addition, GABA-induced colon cancer cells were treated with MG132, resulting in increased cMYC expression. However, MG132 did not significantly increase the expression of cMYC in CBLB knockdown colon cancer cells. While MG132 treating CBLB overexpressed colon cancer cells, it showed consistent results (Fig. S 9F, G). It indicates that CBLB regulates the degradation of cMYC through a proteasome-dependent pathway. We further explored the polyubiquitinated form of endogenous cMYC in CBLB knockdown colon cancer cells. In the presence of MG132, the ubiquitination form of endogenous cMYC protein in CBLB knockdown group was significantly reduced (Fig. 8H). To investigate further mechanism CBLB regulates cMYC protein levels, we explored whether CBLB directly binds to cMYC. Endogenous immunoprecipitation demonstrated that CBLB protein binds to cMYC (Fig. 8I, S9H). Immunofluorescence analysis of SW480 and LoVo cells revealed colocalization of CBLB and cMYC in cells (Fig. 8J). These results revealed that CBLB, as an E3 ligase, can recognize and bind the substrate cMYC. It promotes the ubiquitination of cMYC protein, which destroys the stability of cMYC protein and allows it to be degraded by proteasome.To determine the key domains responsible for this interaction, three HA-labeled CBLB truncations were generated based on previous studies on the structure of CBLB, and the interaction of each truncation with cMYC was examined (Fig. S10A). The amino acids 344–930 and 931–982 were responsible for the interaction with cMYC (Fig. S10B). Similarly, three cMYC truncations with Flag tags were designed and tested for their interaction with CBLB (Fig. S10C). The results confirmed that the amino acid 369–421 was vital in maintaining this interaction (Fig. S10D).

Our data suggests that miR-223-3p can target E3 ligase CBLB, and CBLB protein can interact with cMYC to promote its ubiquitination degradation. MiR-223-3p negatively regulates the expression of CBLB and reduces the ubiquitination degradation of cMYC, thus enabling the proliferation and migration of colon cancer cells.

### GABA promotes proliferation and migration of CRC via miR-223-3p/CBLB/cMYC axis

**Fig. 9:**
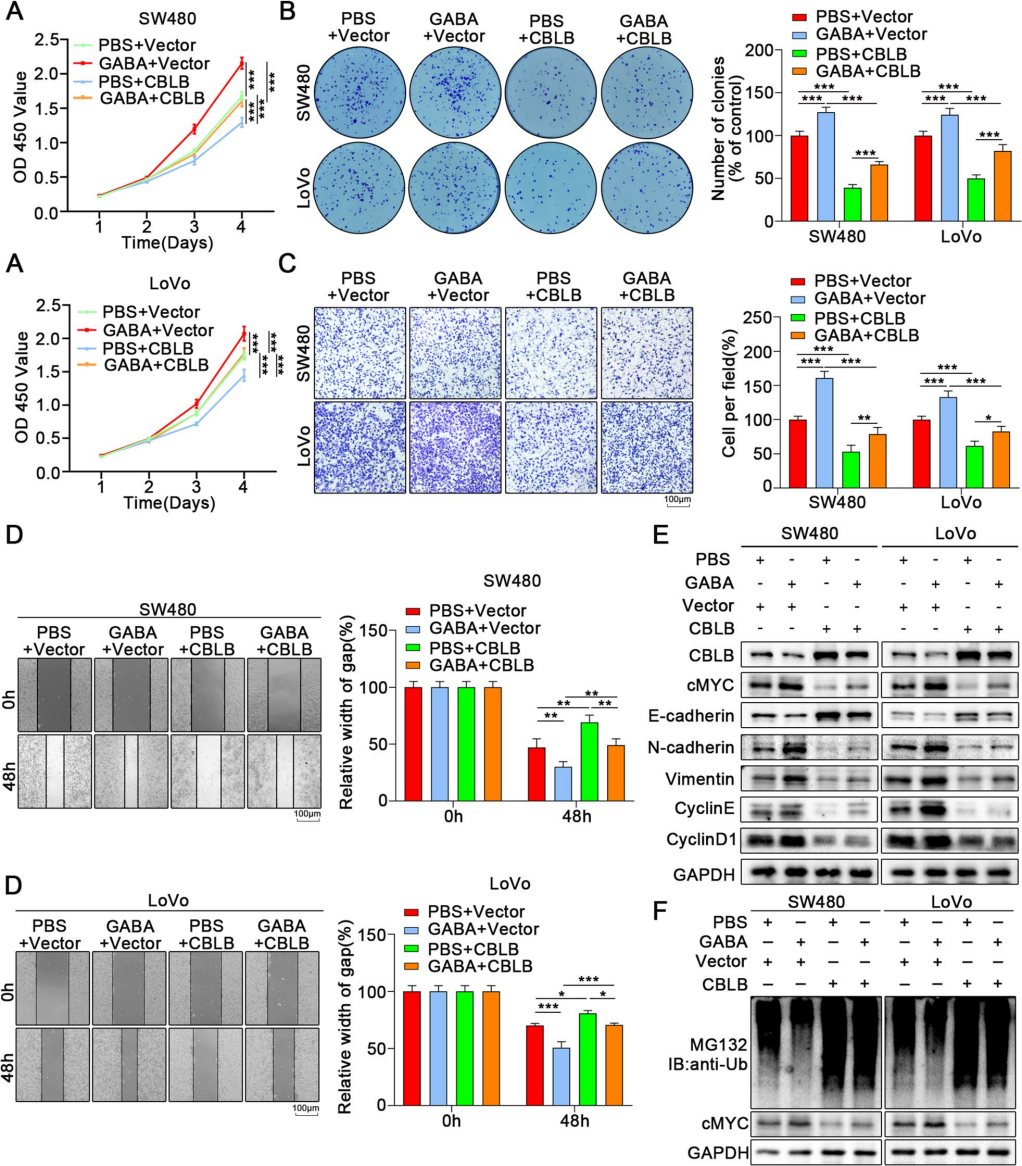
GABA promotes proliferation and migration of CRC via mir-223-3p/CBLB/cMYC axis. **A** CCK-8 assays showed that overexpression of CBLB reversed the effect of GABA on the promotion of proliferation in colon cancer cells. **B** The colony formation assay indicated that overexpression of CBLB reversed the effect of GABA on the promotion of proliferation in colon cancer cells. **C** The transwell assays showed that overexpression of CBLB reversed the effect of GABA on the promotion of migration in colon cancer cells. **D** The wound healing assays showed that overexpression of CBLB reversed the effect of GABA on the promotion of migration in colon cancer cells. **E** Western blot indicated that overexpression of CBLB reversed the effects of GABA on the promotion of proliferation and migration in colon cancer cells. **F** Ubiqutin assays indicated that overexpression of CBLB blocked the altered ubiquitination of cMYC in colon cancer induced by GABA. All data were revealed as mean ± standard deviation (SD) for no less than three independent experiments. Significant *P* values showed as ^***^P < 0.001.^**^P < 0.01

Finally, we conducted a rescue experiment to verify the miR-223-3p/CBLB/cMYC axis involvement in mediating the effects of GABA on colon cancer. GABA increased colon cancer cell proliferation and migration by regulating the ubiquitination of cMYC and promoting the expression of miR-223-3p (Fig. 7C-G). Here, we further investigated whether CBLB mediated the regulation of GABA on cMYC. CBLB was more expressed in GABA-induced colon cancer cells. Overexpression of CBLB significantly decreased the expression of cMYC and inhibited the proliferation and migration of colon cancer cells, while GABA was able to rescue the inhibitory effect of overexpression of CBLB on the proliferation and migration of tumor cells (Fig. 9, S11A-D).

**Fig. 10:**
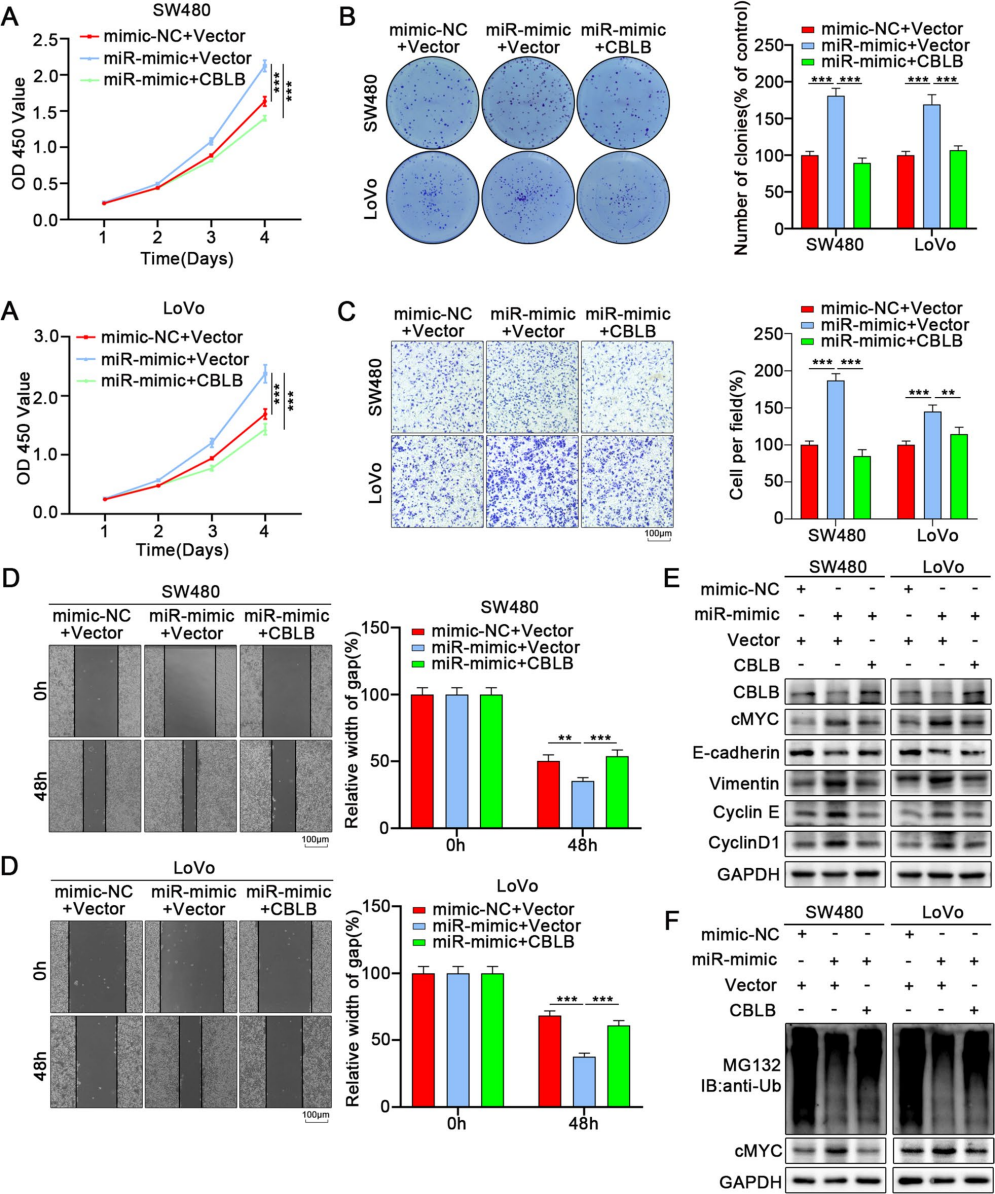
GABA promotes proliferation and migration of CRC via mir-223-3p/CBLB/cMYC axis. **A** CCK-8 assays showed that overexpression of CBLB reversed the effect of miR-223-3p on the promotion of proliferation in colon cancer cells. **B** The colony formation assay indicated that overexpression of CBLB reversed the effect of miR-223-3p on the promotion of proliferation in colon cancer cells. **C** The transwell assays showed that overexpression of CBLB reversed the effect of miR-223-3p on the promotion of migration in colon cancer cells. **D** The wound healing assays showed that overexpression of CBLB reversed the effect of miR-223-3p on the promotion of migration in colon cancer cells. **E** Western blot indicated that overexpression of CBLB reversed the effects of miR-223-3p on the promotion of proliferation and migration in colon cancer cells. **F** Ubiqutin assays indicated that overexpression of CBLB blocked the altered ubiquitination of cMYC in colon cancer induced by miR-223-3p. All data were revealed as mean ± standard deviation (SD) for no less than three independent experiments. Significant *P* values showed as ^***^P < 0.001.^**^P < 0.01

**Fig. 11:**
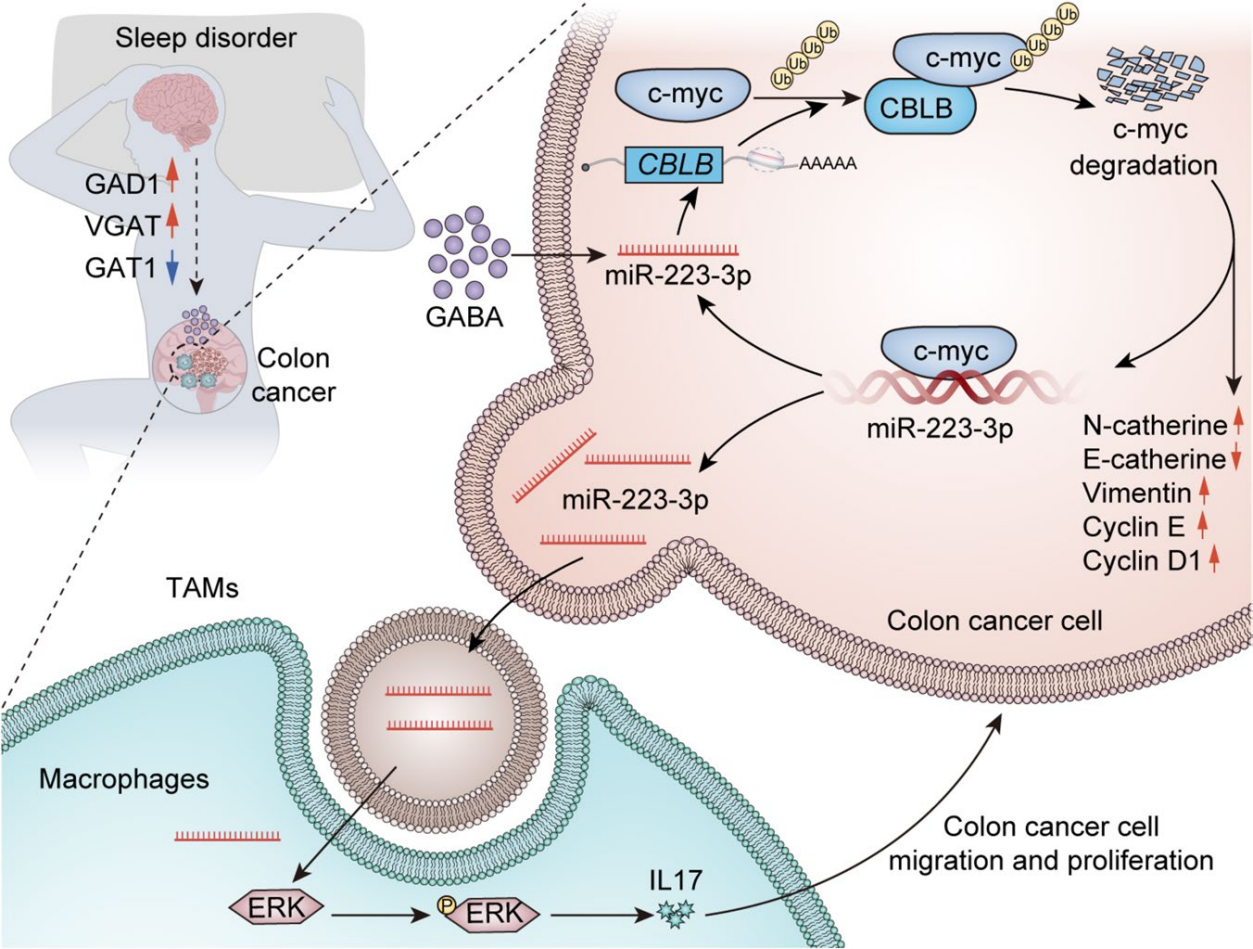
The schematic diagram demonstrated GABA induced by sleep deprivation promotes the proliferation and migration of colon cancer cells through two pathways of miR-223-3p

We further explored the effect of the miR-223-3p/CBLB/cMYC axis on colon cancer by another rescue experiment. We transfected mimics into SW480 and LoVo cells and then transfected the CBLB overexpression plasmid. The results demonstrated that overexpression of CBLB could reverse the promotion of mimics on the proliferation and migration of tumor cells (Figs. 10 and 11, S11E). Immunoprecipitation results revealed that overexpression of miR-223-3p enhanced the stability of cMYC and reduced the ubiquitinated degradation of cMYC, while overexpression of CBLB reversed this effect (Fig. 9E). In addition, we also transfected inhibitor into CBLB knockdown tumor cells, and the results of WB analysis and immunoprecipitation also suggested the same results (Fig. 9F). The development of the rescue function experiment is also consistent with our conclusion (Fig. S11, F).

## Discussion

Sleep is mandatory for maintaining and prolonging human health, and excessive evidence highlights the importance of sleep deprivation in the occurrence and development of tumors. Indeed, individuals with tumors often experience sleep disorder due to factors such as tumor burden, treatment plans, psychological stressors, and more [42]. The secretion of IL-1β by tumor cells emerges as a contributing factor to the disruption of REM sleep and subsequent sleep deprivation by affecting the levels of various sleep-related neurotransmitters, including prostaglandins, nitric oxide, GABA, and others [42]. These establish a self-perpetuating cycle between sleep disorder and tumor circuit, undoubtedly.

For example, Jeremy, et al. reported that non-metastatic breast cancer in mice led to sleep disruption and fragmentation through hypothalamic secretory/orexin (HO) activation, promoting liver glucose processing induced by the tumor [43]. Another study found intermittent sleep accelerated tumor growth and invasion by recruiting TAMs and activating the TLR4 signaling pathway [44]. In our study, the data demonstrated that sleep disorder promoted the growth and migration of colon cancer while noting increased recruitment of macrophages in the lung metastasis tissues of sleep-deprivated mice.

The transition between wakefulness and sleep depends on hypothalamic and brainstem regulation [42]. Neurons are sensitive to changes in peripheral signals such as leptin, cytokines, glucose, amino acids, and pH and regulate the circadian rhythm through the hypothalamus, pituitary-adrenal axis, and sympathetic nervous system [3]. The specific inputs from cholinergic, GABAergic, and 5-HT neurons indicate that these neurons play an important role in endocrine, arousal, and metabolic functions [45–47]. GABA is a non-protein amino acid with high concentrations in different brain regions [48]. It has been reported that sleep-related neurons in the ventrolateral preoptic area contain inhibitory neurotransmitters GABA and alanine and dominate other components of the ascending wake-up system [49].

Our data concluded that sleep inhibition triggers active GABA synthesis in brain tissue, elevates GABA-specific transporter expression, and significantly increases GABA content in peripheral blood. It is known that GABA regulates tumor proliferation and migration as a tumor signal molecule [10]. Our study found that sleep deprivation facilitate the proliferation and migration of colon cancer cells by secreting substantial GABA into the peripheral blood.

CMYC is one of human tumors’ most frequently activated oncoproteins [24]. In recent years, studies have shown that abnormal activation of cMYC determines the pathology and phenotype of tumors and promotes tumor cell immune escape [50]. It has been reported that the inactivation of cMYC can lead to the continuous regression of tumors in clinical models [24]. Therefore, ubiquitination regulation is a crucial anti-tumor approach targeting cMYC. Wang et al. reported that the E3-linked enzyme MAGI3 can regulate ubiquitination degradation of cMYC protein in colon cancer cells, restraining cell growth and enhancing apoptosis [28]. Another study revealed that LncRNA GLCC1 inhibited the ubiquitination of cMYC, stabilizing its protein and promoting the development of colon cancer and tumor metabolism [51]. We found that GABA can increase the stability of the cMYC protein in colon cancer cells, promoting tumor cell proliferation and migration by inhibiting the ubiquitination degradation of cMYC.

In our previous experiments, GABA-induced lung metastasis of colon cancer cells was more pronounced, and more recruitment of associated macrophages were observed in the lung metastasis tissues. Exosomes are the main components of extracellular vesicles (EV), and their diameters range from 30 to 150 nm [14]. Recent studies have claimed that exosomes can transfer DNA, RNA, or proteins within TME to facilitate communication between tumor cells and promote tumor metastasis [52, 53].

MicroRNAs are non-coding RNA of about 20-25nt in length, abundant in exosomes, and play a vital role in intercellular communication [54, 55]. Zhao. Et al. reported that colon cancer-derived exosome miR-934 activates the PI3K/AKT pathway to induce macrophage M2 polarization and promote liver metastasis of colon cancer through CXCL13 and other cytokines [56]. He et al. found that in ovarian cancer, highly expressed miR-205 is transported via exosomes, promoting angiogenesis and distant metastasis through the PTEN/AKT pathway [57]. However, the mechanism of exosomes in the mediating communication between colon cancer cells and TAMs remains unclear, and whether GABA affects the aforementioned intercellular communication is still unknown. Our data suggests that GABA alone has minimal influence on macrophage polarization, but GABA-induced colon cancer cells significantly promote M2 polarization of macrophages during co-culture.

MiR-223-3p regulates tumorigenesis through direct control over tumor cells and indirect regulation via the tumor microenvironment. For example, miR-223 directly targets SEPT6 to inhibit cell apoptosis, promoting migration and invasion in prostate cancer [40]. In addition, HPV infection promotes cervical cancer progression by upregulating the miR-223 expression in cervical tissue, thereby reducing cell adhesion. Recent studies have outlined the endogenous expression of miR-223-3p and its transfer to surrounding target cells through exosomes or extracapsular vesicles, facilitating its biological functions [58]. Zhu et al. confirmed that macrophages transport miR-223 to epithelial ovarian cancer cells via exosomes, promoting the chemotherapy resistance of tumors [59]. Similarly, neutrophils transport miR-223 to lung cancer cells through extracellular vesicles, activating epithelial-mesenchymal transition [60].

Our data indicates that sleep deprivation upregulates the level of GABA in peripheral blood. Furthermore, the result of miRNA sequencing suggests that GABA promotes the expression of miR-223-3p in colon cancer cells. Interestingly, Marçola M, et al. reported that there is a significant difference in the expression of some miRNAs in primary cells extracted during the day and night. Among them, miR-223-3p is highly expressed in nighttime cells [61]. This result suggests that miR-223-3p may be regulated by the diurnal rhythm and play an important role in the process of sleep deprivation promoting colon cancer. Additionally, we found that miR-223-3p can induce M2 polarization of macrophages via the exosome pathway. In turn, macrophages overexpressing miR-223-3p promote colon cancer cell proliferation and migration by secreting IL-17.

MiRNAs usually perform their functions by binding to the 3 'UTR region of the target gene [62]. Our previous study demonstrated that GABA inhibits the ubiquitination of cMYC and promotes colon cancer cell proliferation and migration. Studies have shown that miR-223-3p can regulate protein ubiquitination by regulating E3 ligase expression. Wang et al. reported that FBX8, as the target gene of miR-223-3p, promotes colon cancer proliferation and invasion by mediating the ubiquitination of mTOR [63]. In acute lymphoblastic leukemia, miR-223-3p mediated by the Notch pathway inhibits the E3 ligase FBXW7 expression [64]. Our study found that overexpressing miR-223-3p in colon cancer cells also inhibits the ubiquitination degradation of cMYC, mirroring the effect observed with GABA. Additionally, miR-223-3p overexpression enhanced colon cancer cell proliferation and migration, thus further mediating miR-223-3p in regulating GABA on cMYC protein and promoting colon cancer metastasis.

CBLB is a ring finger type E3 ligase member that mediates various signal proteins' ubiquitination [65]. It has been reported that CBLB combined and ubiquitinates the inflammasome NLRP3, leading to its proteasome degradation and migrating endotoxemia [66]. Similarly, Hong et al. reported that CBLB mediates the lysosomal degradation of activated EGFR through k63-linked ubiquitination in lung adenocarcinoma [67]. However, studies have suggested that the higher CBLB expression is related to the development of several malignant tumors, such as breast cancer [68], melanoma [69], head and neck cancer [70], and more. Furthermore, studies have confirmed that CBLB knockout or deletion can promote the immune activation of CD8 + T cells [71] and enhance the cytotoxicity of NK cells in cancer immunotherapy [72].

However, the role of CBLB in colon cancer remains unclear. This study uncovered that the CBLB expression in colon cancer tissues is low and negatively correlated with their proliferation and migration. Additionally, we found that miR-223-3p downregulates the CBLB expression in colon cancer cells, inhibiting the ubiquitination degradation of cMYC protein via CBLB binding. Our data suggests that GABA induces miR-223-3p overexpression, thereby enhancing the stability of cMYC protein in colon cancer cells by downregulating CBLB expression. Moreover, chip assay and DNA gel analysis showed that cMYC, functioning as a transcription factor, binds to the miR-223-3p promoter, promoting its transcription in colon cancer cells.

## Conclusions

In conclusion, our study discovered a novel mechanism through which sleep deprivation promote the occurrence and development of colon cancer via GABA release. Within colon cancer cells, miR-223-3p inhibits ubiquitination and proteasomal degradation of cMYC through endogenous regulation. Furthermore, miR-223-3p also participates in the intricate communication between colon cancer cells and macrophages through exosomes, further promoting colon cancer cell proliferation and migration. Improving sleep is still the primary priority of cancer prevention and suppression. However, the mechanism of sleep is complex, and the interplay impact between sleep and tumors necessitates further exploration. Based on current research, targeting GABA levels and miR-223-3p expressions emerges as a potential therapeutic strategy to treat colon cancer and slow down tumor progression. Additionally, a deeper investigation into GABA and miR-223-3p roles in the TME is needed to clarify the precise molecular mechanism underpinning cell-to-cell communication in the TME.

### Supplementary Information

**Additional file 1:** **Figure. S1.** Sleep disorders promotes occurrence and metastasis of CRC by GABA. (A-C) ELISA assay showed the level of NE, EPI and 5-HT in the serum of SD group and control group mice. (D) Schematic illustration of the AOM/DSS model mice experiment design. PBS or GABA intraperitoneal injection twice every week. (E) Macroscopic images of AOM/DSS-induced colonic tumors in PBS group and GABA group mice.

**Additional file 2:** **Figure. S2.** GABA inhibits ubiquitination of cMYC and promotes proliferation and migration of CRC.  (A) A cluster heatmap of expression of GABARAP, GABBR1 and GABBR2 in 30 paired samples from GSE database. (B) The expression of GABARAP and GABBR2 in paired colon cancer tissue and paracancer tissue was detected by qRT-PCR. (**C**) The expression of GABBR2 in HCT116, SW480, LoVo and DLD1 cells was detected by Western blot. (D) The expreesion of E-cadherin, N-cadherin, Vimentin, CyclinE and Cyclin D1 in SW480 and LoVo cells treated by different concentration of GABA was detected by Western blot. (E) Western blot revealed that GABA promoted the proliferation and migration in SW480 and LoVo cells. (F) The wound healing assays showed that cMYC knockdown reversed the effects of GABA-induced promotion of migration in colon cancer cells.

**Additional file 3:** **Figure. S3.** GABA promotes the recruitment of macrophages by CRC and induces M2 polarization through exosomes. (A) Transwell assays revealed that co-culture with GABA-induced colon cancer cells increased the migrative ability of THP1 cells. (B) Representative image of macrophages derived from THP1 cells treated with phorbol 12-myristate 13-acetate (PMA) for 24h. (C) The expression of macrophage marker CD86 in THP1 cells was detected by qRT-PCR. (D) The expression of CD206, CD163, Arginase1 and CD86 of THP1 cells co-cultured with GABA-induced LoVo or not was detected by qRT-PCR. (E-F) Western blot and qRT-PCR showed that the expression of M2 makers (CD206, CD163, Arginase1) in THP1 cells was increased more significantly when added GABA-induced SW480-CM by qRT-PCR, while M1 maker (CD86) was decreased. (G) Western blot was performed to detect typical exosomal biomarkers (TSG101, CD9) in exosomes derived from colon cancer cells treated by GABA or PBS. (H-I) Phenotype analysis of exosomes derived from colon cancer cells treated by GABA or PBS using electron microscopy and Nano Sight nanoparticle tracking analysis. (J) Immunofluorescence image showed that THP1 cells exhibited more pronounced M2 polarization when incubated GABA-induced LoVo-derived exosomes for 3 days. (K) Western blot showed that GW4869 (an inhibitor of exosome secretion) reversed the strengthened M2 polarization of THP1 cells upon incubated GABA-induced SW480-derived exosomes.

**Additional file 4:** **Figure. S4.** Exosome miR-223-3p promotes M2 polarization of macrophages, which aggravates the proliferation and migration of CRC. (A) The expression of miRNAs which were predicted upregulated in Exosome sequencing in LoVo cells treated by GABA or PBS was detected by qRT-PCR. (B) qRT-PCR was performed to detect the expression of miR-150-5p and miR-223-3p in CM, Exosomes depleted CM and exosomes respectively, which were derived from LoVo treated by GABA or PBS. (C) KEGG analysis about predicted target genes of upregulated miRNAs in GABA-induced colon cancer cells-derived exosomes. (D) Western blot showed that inhibition of the ERK pathway effectively reversed the M2 polarization of macrophages induced by miR-223-3p.SB-203580: p38 MAPK inhibitor. SCH-772984: ERK MAPK inhibitor. (E) The proliferation of LoVo cells co-cultured with THP1 overexpressed miR-223-3p or not was assessed via CCK8 for 3 days. (F) The proliferation of LoVo cells co-cultured with THP1 cells overexpressed miR-223-3p or not was assessed via colony formation assay for 10 days. (G).The transwell assays indicated that co-cultured with THP1 cells overexpressed miR-223-3p increased the migrative ability of LoVo cells. (H) The wound healing assays showed that co-cultured with THP1 cells overexpressed miR-223-3p increased the migrative ability of LoVo cells.All data were revealed as mean ± standard deviation (SD) for no less than three independent experiments. Significant *P*values showed as ^***^P <0.001.^**^P<0.01.^*^P <0.05. ns means the difference was not significant.

**Additional file 5:** **Figure. S5.** Exosome miR-223-3p promotes M2 polarization of macrophages, which aggravates the proliferation and migration of CRC. (A-C) Co-culture with THP1 cells overexpressed miR-223-3p increased the volume and weight of subcutaneous tumors. (D-E) The number of tumors in the lung was counted upon co-cultured with THP1 cells overexpressed miR-223-3p or not. (F) HE staining showed the tumors in the lung of mice. (G) Western blot indicated that the proliferation and migration of colon cancer cells was increased when co-culture with THP1 cells overexpressed miR-223-3p. (H) The wound healing assays showed that migrative ability was significantly augmented when added the CM of THP1 cells overexpressed miR-223-3p. (I) Western blot indicated that the proliferation and migration of colon cancer cells was increased when added the CM of THP1 cells overexpressed miR-223-3p. All data were revealed as mean ± standard deviation (SD) for no less than three independent experiments. Significant *P *values showed as ^***^P <0.001.^**^P<0.01.

**Additional file 6:** **Figure. S6.** GABA promotes the proliferation and migration of CRC by inhibiting ubiquitination of cMYC through miR-223-3p. (A) The proliferation of colon cancer cells transfected with miR-mimic or negative control was assessed via CCK8 for 3 days. (B) The proliferation of colon cancer cells transfected with miR-mimic or negative control was assessed via colony formation assay for 10 days. (C) The wound healing assays showed that overexpression of miR-223-3p augmented the migrative ability of colon cancer cells. (D) The transwell assays indicated that overexpression of miR-223-3p augmented the migrative ability of colon cancer cells. (E) qRT-PCR showed that the expression of miR-223-3p was significantly increased when overexpressed cMYC in LoVo cells. (F) qRT-PCR showed that the expression of miR-223-3p was significantly decreased when knocked down cMYC in LoVo cells. (G) The schematic diagram exhibited one predicted binding site between cMYC and the miR-223-3p promoter. (H) Chip assays with cMYC antibody or IgG were performed to verify binding between cMYC and the miR-223-3p promoter in 293T cells. All data were revealed as mean ± standard deviation (SD) for no less than three independent experiments. Significant *P *values showed as ^***^P<0.001.^*^P <0.05.

**Additional file 7:** **Figure. S7.** Mir-223-3p targets E3 ligase CBLB to regulate ubiquitination of cMYC. (A)The expression of CBLB in paired colon cancer tissue and paracancer tissue was detected by Western blot. (B) The expression level of CBLB was detected by IHC in paired colon cancer tissue and non-cancerous tissue. (C) Western blot showed that sleep deprivation downregulates the expression of CBLB in subcutaneous tumor of colon cancer, while the expression of cMYC is upregulated significantly. (D) The proliferation of colon cancer cells upon CBLB overexpression or knockdown was assessed via CCK8 for 3 days. (E) The proliferation of colon cancer cells upon CBLB overexpression or knockdown was assessed via colony formation assay for 10 days. (F) The transwell assays indicated that overexpression of CBLB decreased the migrative ability of colon cancer cells. All data were revealed as mean ± standard deviation (SD) for no less than three independent experiments. Significant *P *values showed as ^***^P<0.001.^**^P <0.01.

**Additional file 8:** **Figure. S8.** MiR-223-3p targets E3 ligase CBLB to regulate ubiquitination of cMYC. (A) The proliferation of LoVo cells upon CBLB overexpression or knockdown was assessed via CCK8 for 3 days. (B-C) The wound healing assays showed that overexpression of CBLB decreased the migrative ability of colon cancer cells. All data were revealed as mean ± standard deviation (SD) for no less than three independent experiments. Significant *P *values showed as ^***^P <0.001.^**^P <0.01.^*^P<0.05.

**Additional file 9:** **Figure. S9.** MiRr-223-3p targets E3 ligase CBLB to regulate ubiquitination of cMYC. (A). Immunofluorescence assay indicated that knockdown CBLB increased mesenchymal markers of colon cancer cells but reduced epithelial markers. (B-D) CBLB knockdown increased the volume and weight of subcutaneous tumors, while overexpression of CBLB decreased the volume and weight of subcutaneous tumors. (E) Colon cancer cells were knocked down CBLB followed by treatment with cycloheximide (CHX) for the indicated times. The intensity of cMYC expression at each time point was quantified by densitometry and plotted against time. (F) Western blot was performed to detected the expression of cMYC in colon cancer cells which were knocked down CBLB and then incubated with or without MG132 for 6h. (G) Western blot was performed to detected the expression of cMYC in colon cancer cells which were overexpressed CBLB and then incubated with or without MG132 for 6h.(H) CO-IP and Western blot showed that endogenous cMYC and CBLB bind to each other. All data were revealed as mean ± standard deviation (SD) for no less than three independent experiments. Significant *P *values showed as ^***^P<0.001.

**Additional file 10:** **Figure. S10.** MiR-223-3p targets E3 ligase CBLB to regulate ubiquitination of cMYC. (A-B). Immunoprecipitation of CBLB constructs and cMYC in 293T cells. (C-D). Immunoprecipitation of cMYC constructs and CBLB in 293T cells.

**Additional file 11:** **Figure. S11.** GABA promotes proliferation and migration of CRC via miR-223-3p/CBLB/cMYC axis. A. CCK8 assays showed that CBLB knockdown reversed the effect of inhibitor of miR-223-3p on the inhibition of proliferation in colon cancer cells. B. The colony formation assay indicated that CBLB knockdown reversed the effect of inhibitor of miR-223-3p on the inhibition of proliferation in colon cancer cells. C. The transwell assays showed that CBLB knockdown reversed the effect of inhibitor of miR-223-3p on the inhibition of proliferation in colon cancer cells. D. The wound healing assays showed that CBLB knockdown reversed the effect of inhibitor of miR-223-3p on the inhibition of proliferation in colon cancer cells. E. Western blot indicated that CBLB knockdown reversed the effect of inhibitor of miR-223-3p on the inhibition of proliferation in colon cancer cells. F. Ubiqutin assays indicated that CBLB knockdown blocked the altered ubiquitination of cMYC in colon cancer induced by inhibitor of miR-223-3p. All data were revealed as mean ± standard deviation (SD) for no less than three independent experiments. Significant *P *values showed as ^***^P<0.001.^**^P <0.01.

**Additional file 12:** **Table S1.** Results of miRNAs sequencing.

**Additional file 13:** **Table S2.** The drugs used in this study.

**Additional file 14:** **Table S3.** Primers of genes in this research for qRT‐PCR.

**Additional file 15:** **Table S4.** details of primary antibodies applied in this study.

**Additional file 16:** **Table S5.** Sequence information used in this study.

## Data Availability

The datasets used and/or analysed during the current study are available from the corresponding author on reasonable request.

## Abbreviations

SD: sleep deprivation
CRC: Colorectal cancer
GABA: γ-aminobutyric acid
TME: Tumor microenvironment
PBS: Phosphate-buffered saline
Exo: Exosomes
TAMs: Tumor-associated macrophages
CHX: Cycloheximide
ChIP: Chromatin immunoprecipitation
CBLB: Cbl proto-oncogene B
MAPK: Ras/mitogen-activated protein kinase
cMYC: transcriptional regulator Myc-like
