# Supplementary figures and images for: GABA induced by sleep deprivation promotes the proliferation and migration of colon tumors through miR-223-3p endogenous pathway and exosome pathway
Source: J Exp Clin Cancer Res. 2023 Dec 18;42:344. doi: 10.1186/s13046-023-02921-9 (PMC10726571; doi:10.1186/s13046-023-02921-9)

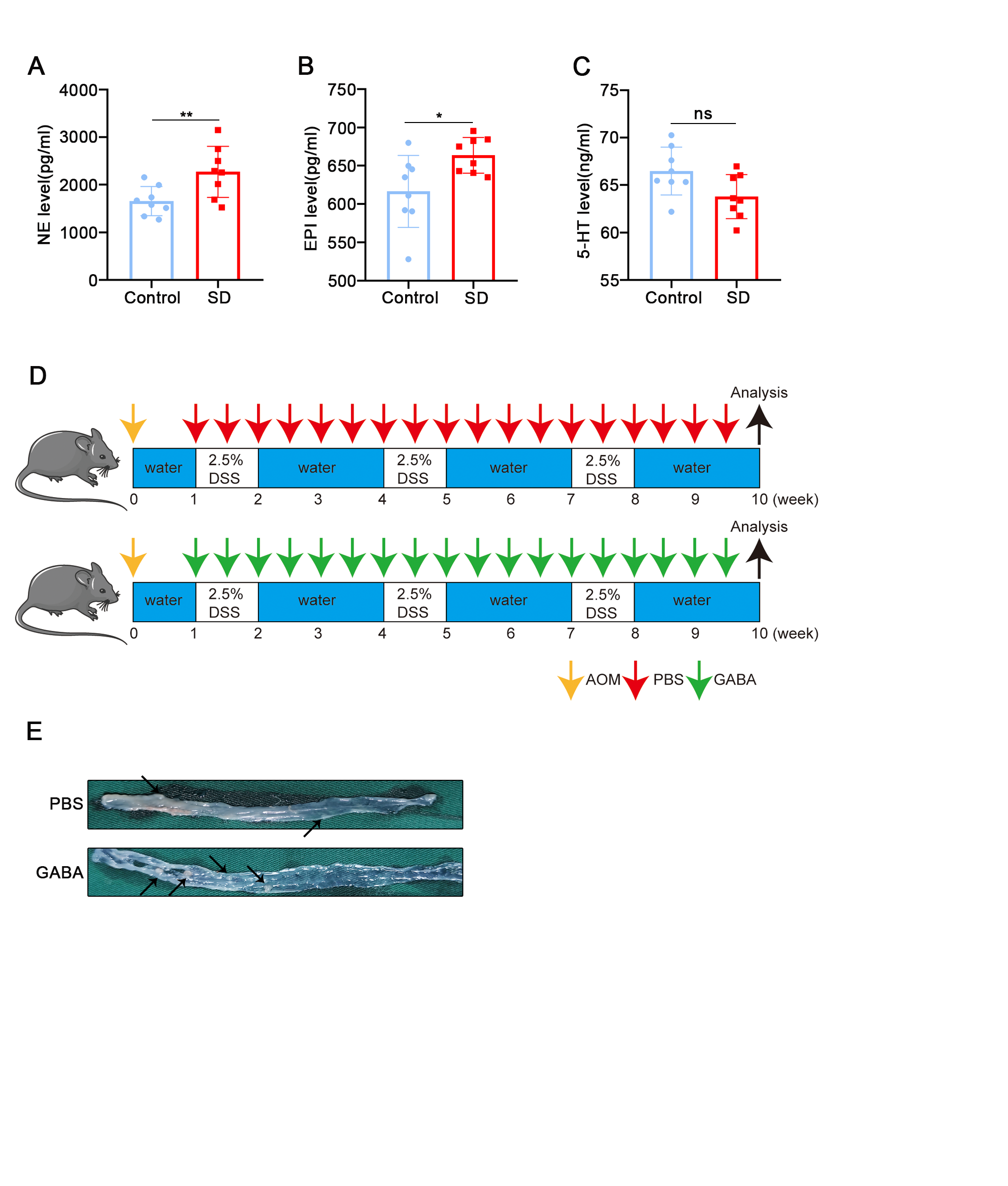

Supplement: Supplementary file 1 — Additional file 1: Figure. S1. Sleep disorders promotes occurrence and metastasis of CRC by GABA. (A-C) ELISA assay showed the level of NE, EPI and 5-HT in the serum of SD group and control group mice. (D) Schematic illustration of the AOM/DSS model mice experiment design. PBS or GABA intraperitoneal injection twice every week. (E) Macroscopic images of AOM/DSS-induced colonic tumors in PBS group and GABA group mice. [file 13046_2023_2921_MOESM1_ESM.png]

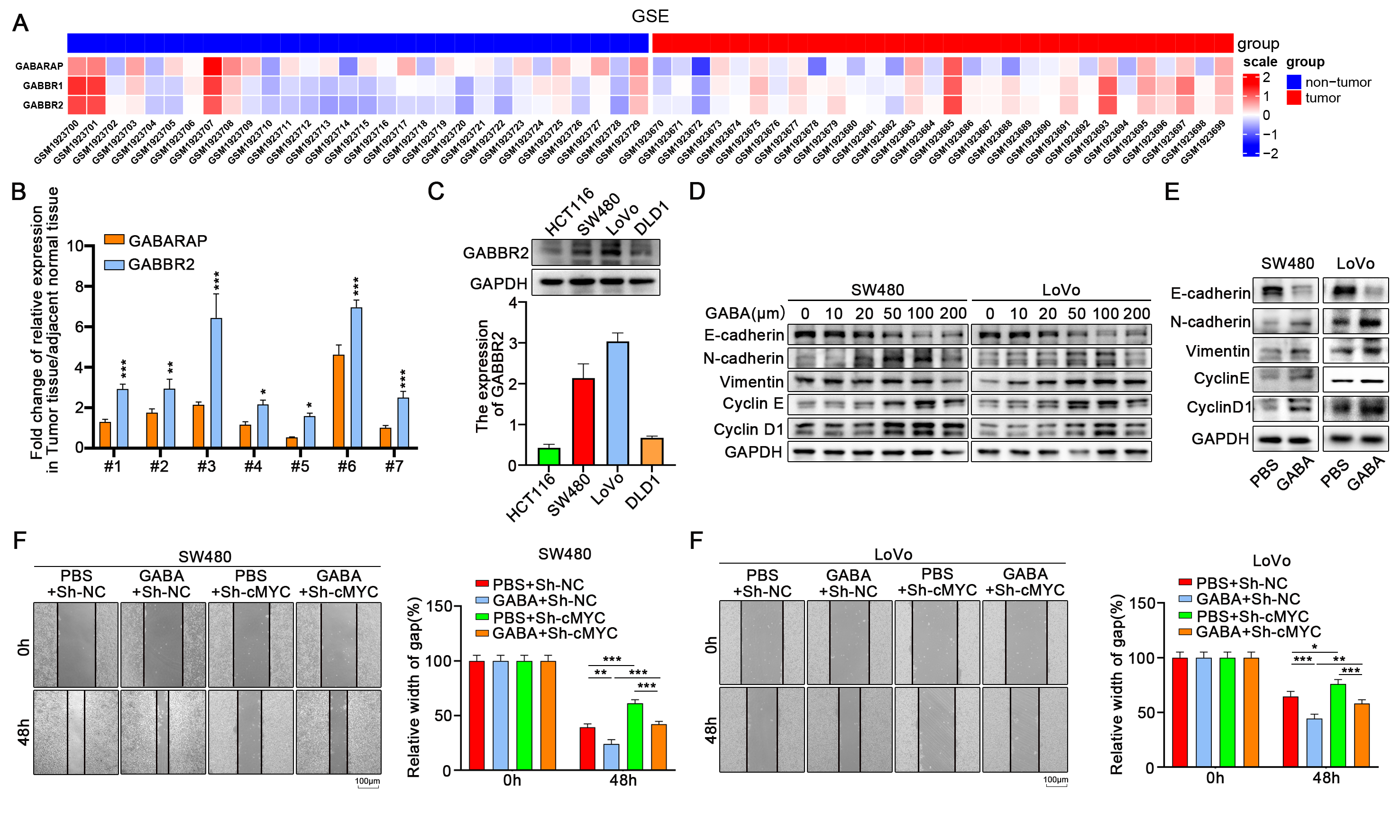

Supplement: Supplementary file 2 — Additional file 2: Figure. S2. GABA inhibits ubiquitination of cMYC and promotes proliferation and migration of CRC. (A) A cluster heatmap of expression of GABARAP, GABBR1 and GABBR2 in 30 paired samples from GSE database. (B) The expression of GABARAP and GABBR2 in paired colon cancer tissue and paracancer tissue was detected by qRT-PCR. (C) The expression of GABBR2 in HCT116, SW480, LoVo and DLD1 cells was detected by Western blot. (D) The expreesion of E-cadherin, N-cadherin, Vimentin, CyclinE and Cyclin D1 in SW480 and LoVo cells treated by different concentration of GABA was detected by Western blot. (E) Western blot revealed that GABA promoted the proliferation and migration in SW480 and LoVo cells. (F) The wound healing assays showed that cMYC knockdown reversed the effects of GABA-induced promotion of migration in colon cancer cells. [file 13046_2023_2921_MOESM2_ESM.png]

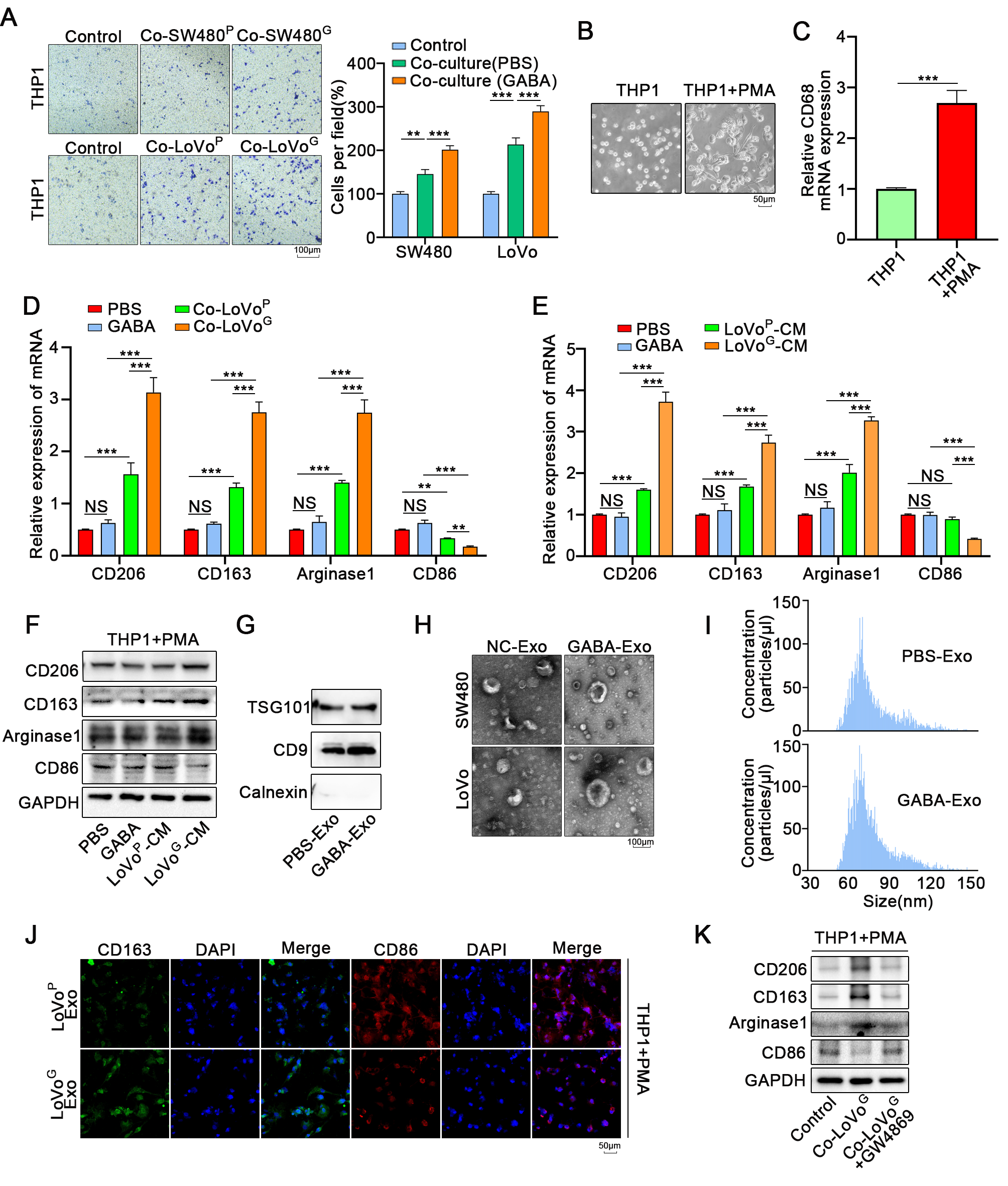

Supplement: Supplementary file 3 — Additional file 3: Figure. S3. GABA promotes the recruitment of macrophages by CRC and induces M2 polarization through exosomes. (A) Transwell assays revealed that co-culture with GABA-induced colon cancer cells increased the migrative ability of THP1 cells. (B) Representative image of macrophages derived from THP1 cells treated with phorbol 12-myristate 13-acetate (PMA) for 24h. (C) The expression of macrophage marker CD86 in THP1 cells was detected by qRT-PCR. (D) The expression of CD206, CD163, Arginase1 and CD86 of THP1 cells co-cultured with GABA-induced LoVo or not was detected by qRT-PCR. (E-F) Western blot and qRT-PCR showed that the expression of M2 makers (CD206, CD163, Arginase1) in THP1 cells was increased more significantly when added GABA-induced SW480-CM by qRT-PCR, while M1 maker (CD86) was decreased. (G) Western blot was performed to detect typical exosomal biomarkers (TSG101, CD9) in exosomes derived from colon cancer cells treated by GABA or PBS. (H-I) Phenotype analysis of exosomes derived from colon cancer cells treated by GABA or PBS using electron microscopy and Nano Sight nanoparticle tracking analysis. (J) Immunofluorescence image showed that THP1 cells exhibited more pronounced M2 polarization when incubated GABA-induced LoVo-derived exosomes for 3 days. (K) Western blot showed that GW4869 (an inhibitor of exosome secretion) reversed the strengthened M2 polarization of THP1 cells upon incubated GABA-induced SW480-derived exosomes. [file 13046_2023_2921_MOESM3_ESM.png]

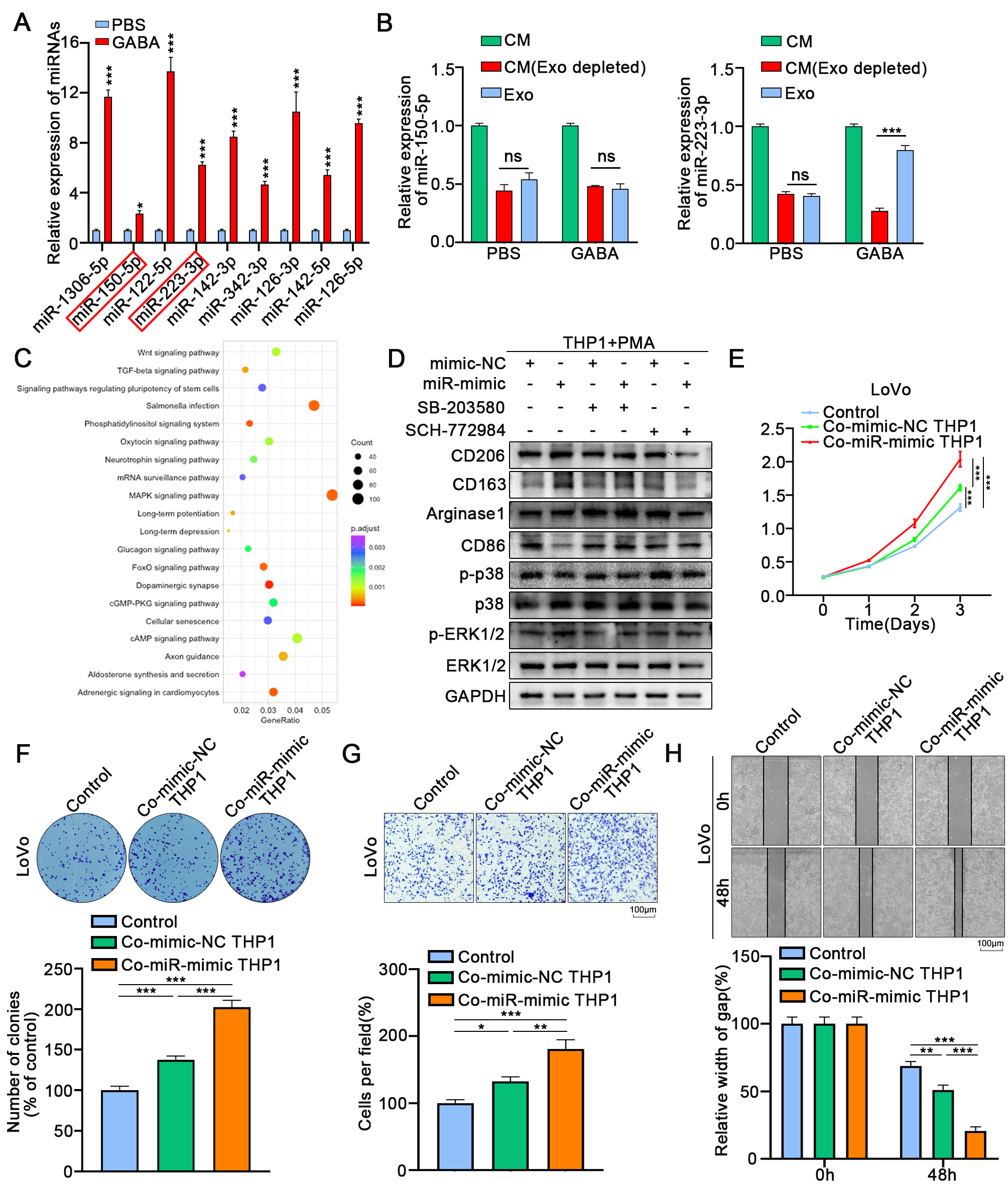

Supplement: Supplementary file 4 — Additional file 4: Figure. S4. Exosome miR-223-3p promotes M2 polarization of macrophages, which aggravates the proliferation and migration of CRC. (A) The expression of miRNAs which were predicted upregulated in Exosome sequencing in LoVo cells treated by GABA or PBS was detected by qRT-PCR. (B) qRT-PCR was performed to detect the expression of miR-150-5p and miR-223-3p in CM, Exosomes depleted CM and exosomes respectively, which were derived from LoVo treated by GABA or PBS. (C) KEGG analysis about predicted target genes of upregulated miRNAs in GABA-induced colon cancer cells-derived exosomes. (D) Western blot showed that inhibition of the ERK pathway effectively reversed the M2 polarization of macrophages induced by miR-223-3p.SB-203580: p38 MAPK inhibitor. SCH-772984: ERK MAPK inhibitor. (E) The proliferation of LoVo cells co-cultured with THP1 overexpressed miR-223-3p or not was assessed via CCK8 for 3 days. (F) The proliferation of LoVo cells co-cultured with THP1 cells overexpressed miR-223-3p or not was assessed via colony formation assay for 10 days. (G).The transwell assays indicated that co-cultured with THP1 cells overexpressed miR-223-3p increased the migrative ability of LoVo cells. (H) The wound healing assays showed that co-cultured with THP1 cells overexpressed miR-223-3p increased the migrative ability of LoVo cells.All data were revealed as mean ± standard deviation (SD) for no less than three independent experiments. Significant Pvalues showed as ***P <0.001.**P<0.01.*P <0.05. ns means the difference was not significant. [file 13046_2023_2921_MOESM4_ESM.png]

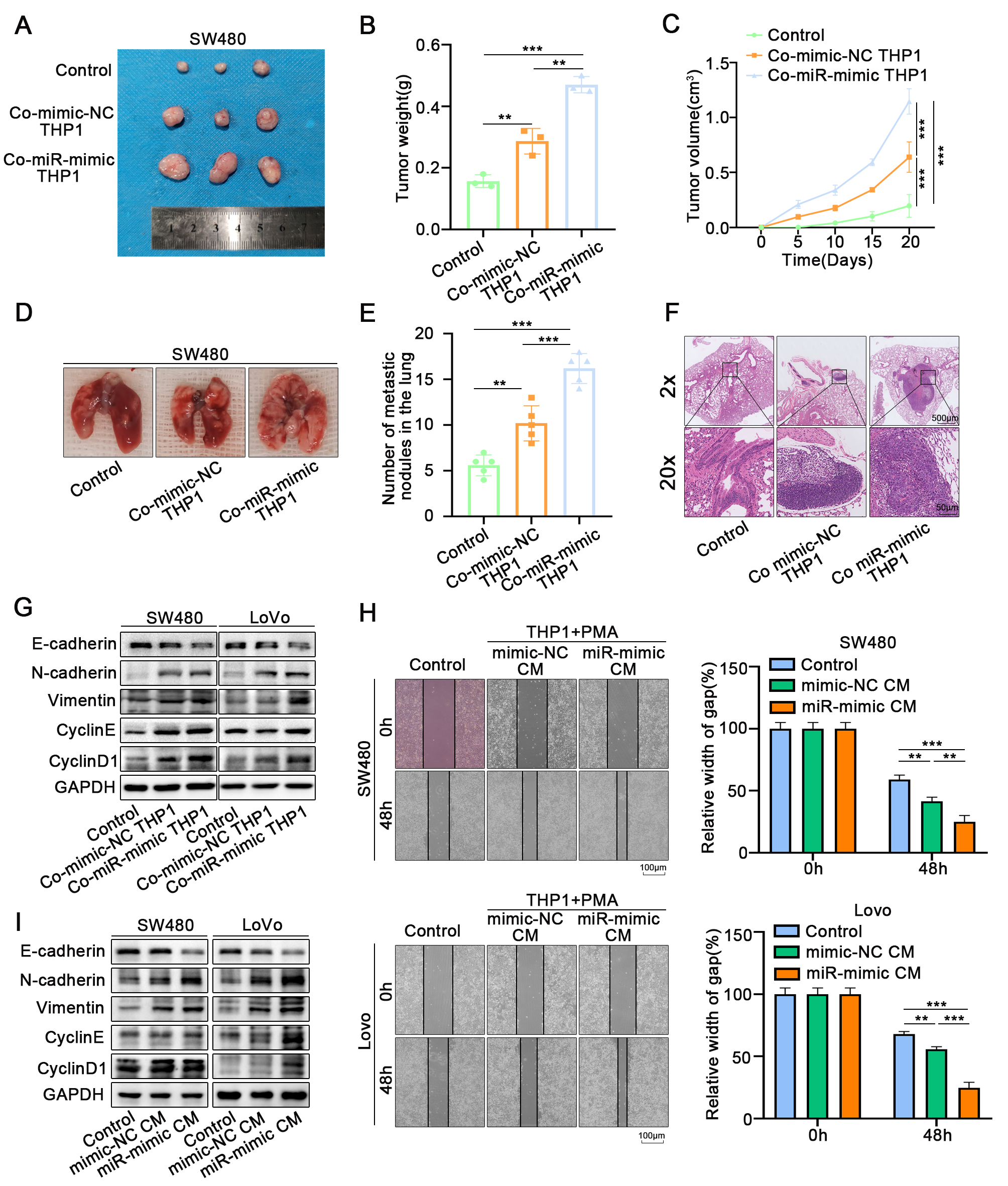

Supplement: Supplementary file 5 — Additional file 5: Figure. S5. Exosome miR-223-3p promotes M2 polarization of macrophages, which aggravates the proliferation and migration of CRC. (A-C) Co-culture with THP1 cells overexpressed miR-223-3p increased the volume and weight of subcutaneous tumors. (D-E) The number of tumors in the lung was counted upon co-cultured with THP1 cells overexpressed miR-223-3p or not. (F) HE staining showed the tumors in the lung of mice. (G) Western blot indicated that the proliferation and migration of colon cancer cells was increased when co-culture with THP1 cells overexpressed miR-223-3p. (H) The wound healing assays showed that migrative ability was significantly augmented when added the CM of THP1 cells overexpressed miR-223-3p. (I) Western blot indicated that the proliferation and migration of colon cancer cells was increased when added the CM of THP1 cells overexpressed miR-223-3p. All data were revealed as mean ± standard deviation (SD) for no less than three independent experiments. Significant P values showed as ***P <0.001.**P<0.01. [file 13046_2023_2921_MOESM5_ESM.png]

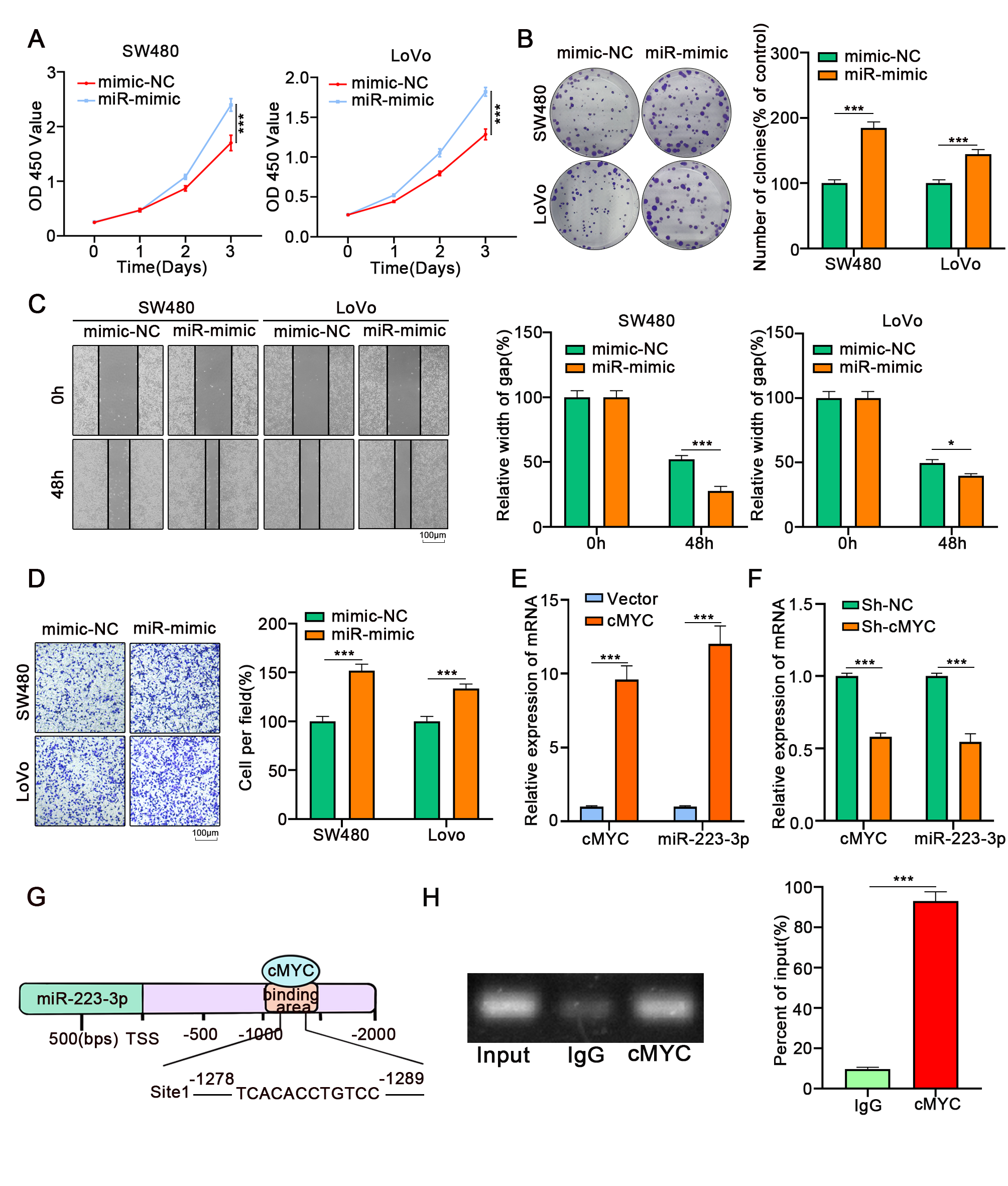

Supplement: Supplementary file 6 — Additional file 6: Figure. S6. GABA promotes the proliferation and migration of CRC by inhibiting ubiquitination of cMYC through miR-223-3p. (A) The proliferation of colon cancer cells transfected with miR-mimic or negative control was assessed via CCK8 for 3 days. (B) The proliferation of colon cancer cells transfected with miR-mimic or negative control was assessed via colony formation assay for 10 days. (C) The wound healing assays showed that overexpression of miR-223-3p augmented the migrative ability of colon cancer cells. (D) The transwell assays indicated that overexpression of miR-223-3p augmented the migrative ability of colon cancer cells. (E) qRT-PCR showed that the expression of miR-223-3p was significantly increased when overexpressed cMYC in LoVo cells. (F) qRT-PCR showed that the expression of miR-223-3p was significantly decreased when knocked down cMYC in LoVo cells. (G) The schematic diagram exhibited one predicted binding site between cMYC and the miR-223-3p promoter. (H) Chip assays with cMYC antibody or IgG were performed to verify binding between cMYC and the miR-223-3p promoter in 293T cells. All data were revealed as mean ± standard deviation (SD) for no less than three independent experiments. Significant P values showed as ***P<0.001.*P <0.05. [file 13046_2023_2921_MOESM6_ESM.png]

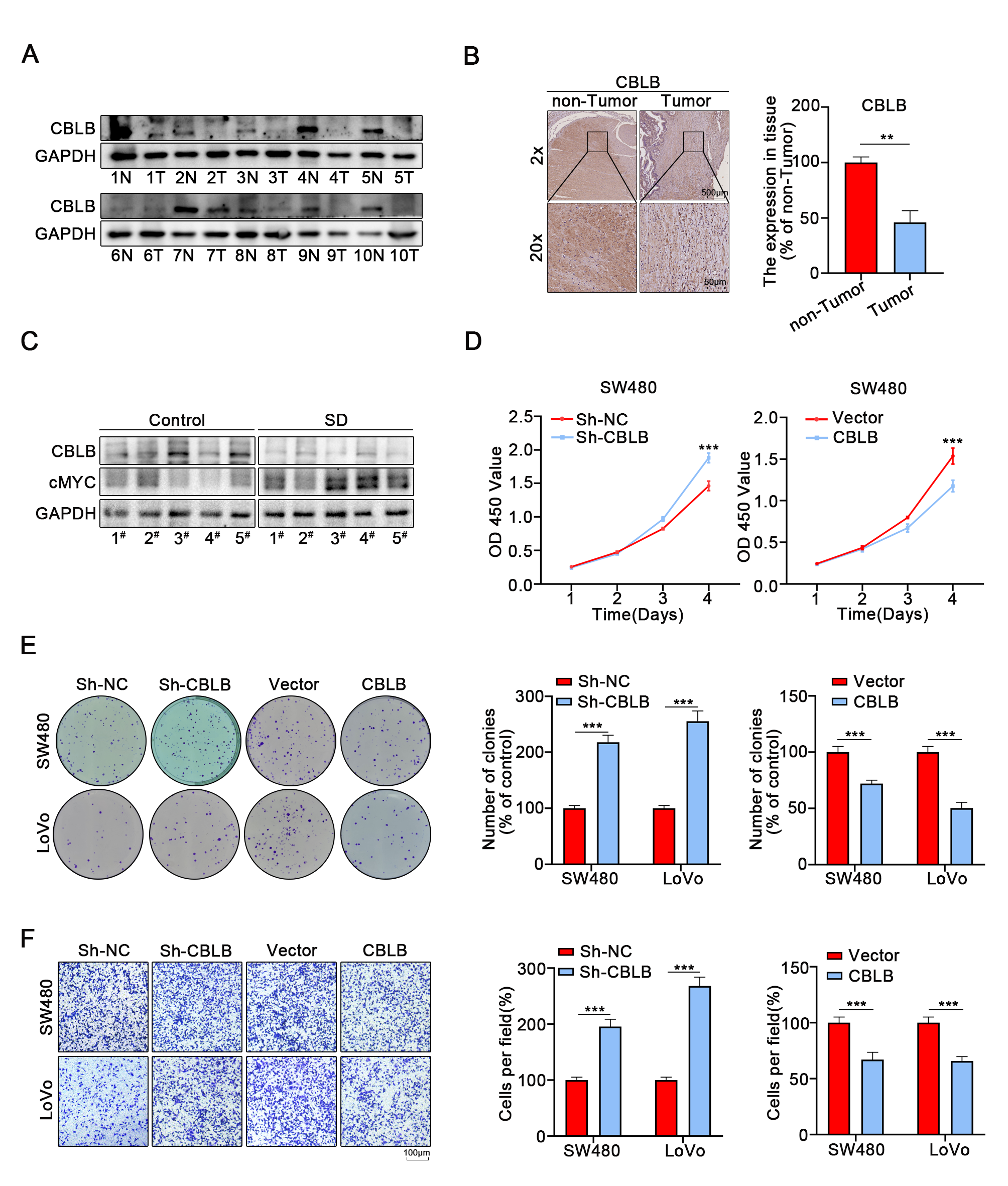

Supplement: Supplementary file 7 — Additional file 7: Figure. S7. Mir-223-3p targets E3 ligase CBLB to regulate ubiquitination of cMYC. (A)The expression of CBLB in paired colon cancer tissue and paracancer tissue was detected by Western blot. (B) The expression level of CBLB was detected by IHC in paired colon cancer tissue and non-cancerous tissue. (C) Western blot showed that sleep deprivation downregulates the expression of CBLB in subcutaneous tumor of colon cancer, while the expression of cMYC is upregulated significantly. (D) The proliferation of colon cancer cells upon CBLB overexpression or knockdown was assessed via CCK8 for 3 days. (E) The proliferation of colon cancer cells upon CBLB overexpression or knockdown was assessed via colony formation assay for 10 days. (F) The transwell assays indicated that overexpression of CBLB decreased the migrative ability of colon cancer cells. All data were revealed as mean ± standard deviation (SD) for no less than three independent experiments. Significant P values showed as ***P<0.001.**P <0.01. [file 13046_2023_2921_MOESM7_ESM.png]

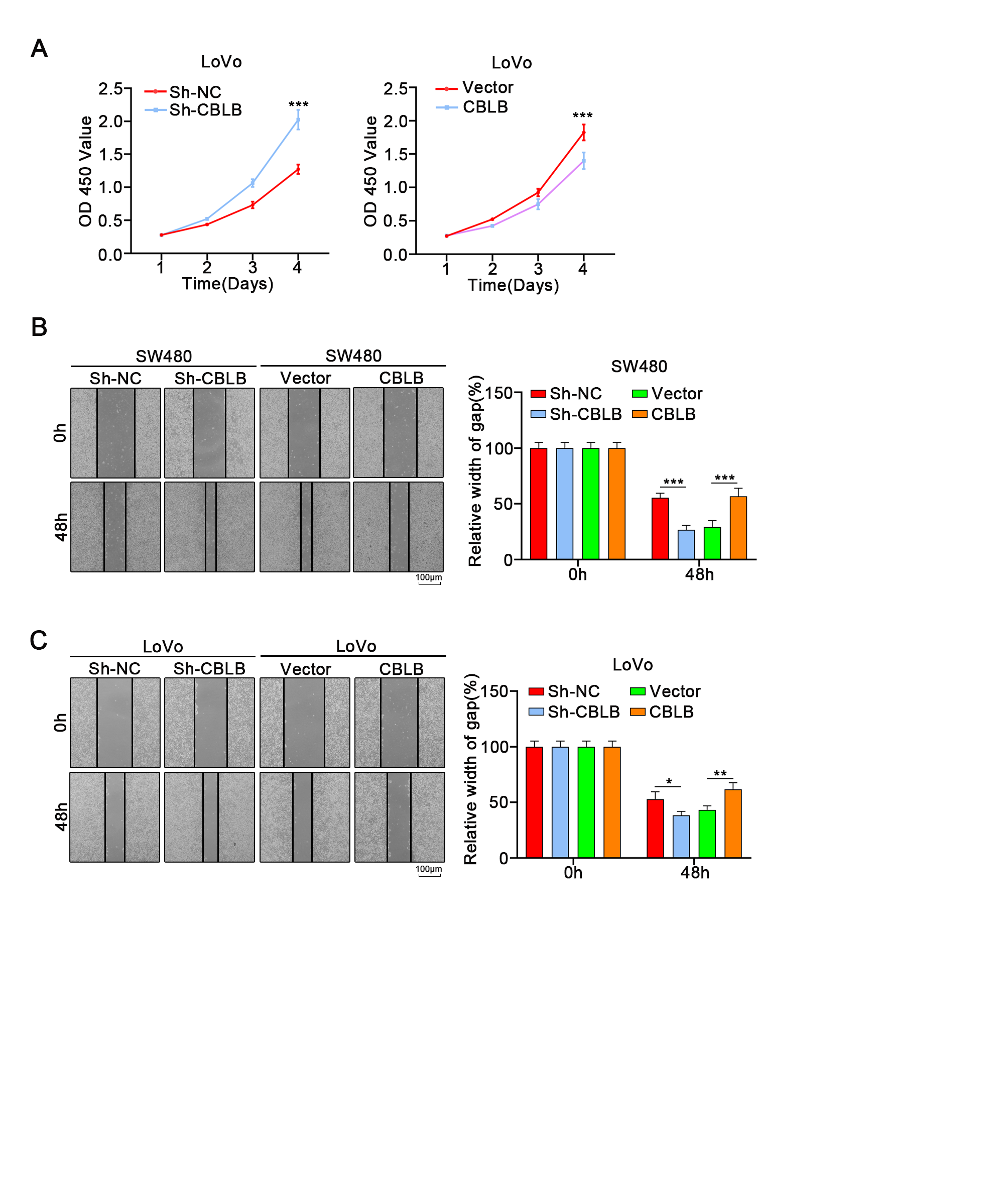

Supplement: Supplementary file 8 — Additional file 8: Figure. S8. MiR-223-3p targets E3 ligase CBLB to regulate ubiquitination of cMYC. (A) The proliferation of LoVo cells upon CBLB overexpression or knockdown was assessed via CCK8 for 3 days. (B-C) The wound healing assays showed that overexpression of CBLB decreased the migrative ability of colon cancer cells. All data were revealed as mean ± standard deviation (SD) for no less than three independent experiments. Significant P values showed as ***P <0.001.**P <0.01.*P<0.05. [file 13046_2023_2921_MOESM8_ESM.png]

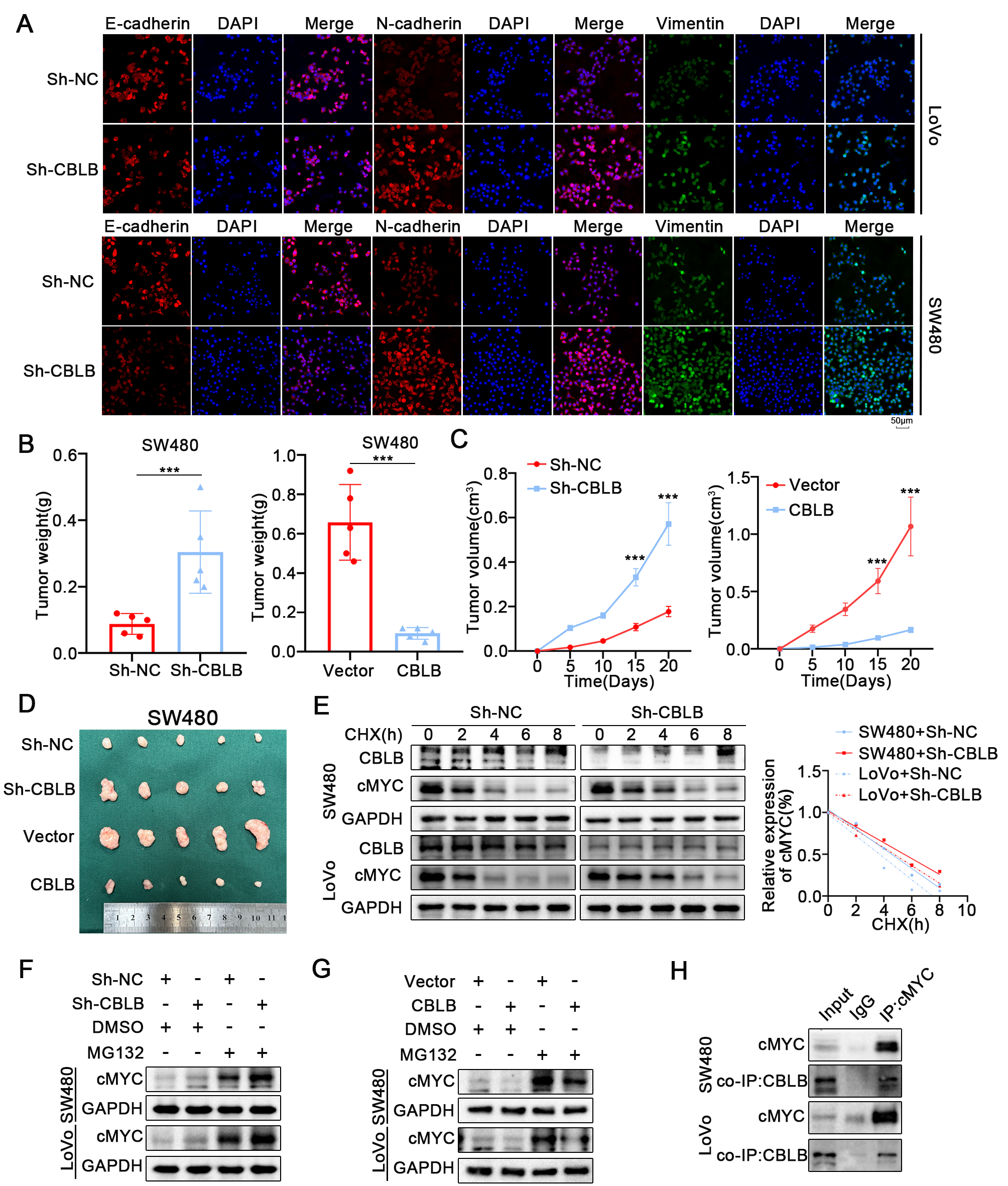

Supplement: Supplementary file 9 — Additional file 9: Figure. S9. MiRr-223-3p targets E3 ligase CBLB to regulate ubiquitination of cMYC. (A). Immunofluorescence assay indicated that knockdown CBLB increased mesenchymal markers of colon cancer cells but reduced epithelial markers. (B-D) CBLB knockdown increased the volume and weight of subcutaneous tumors, while overexpression of CBLB decreased the volume and weight of subcutaneous tumors. (E) Colon cancer cells were knocked down CBLB followed by treatment with cycloheximide (CHX) for the indicated times. The intensity of cMYC expression at each time point was quantified by densitometry and plotted against time. (F) Western blot was performed to detected the expression of cMYC in colon cancer cells which were knocked down CBLB and then incubated with or without MG132 for 6h. (G) Western blot was performed to detected the expression of cMYC in colon cancer cells which were overexpressed CBLB and then incubated with or without MG132 for 6h.(H) CO-IP and Western blot showed that endogenous cMYC and CBLB bind to each other. All data were revealed as mean ± standard deviation (SD) for no less than three independent experiments. Significant P values showed as ***P<0.001. [file 13046_2023_2921_MOESM9_ESM.png]

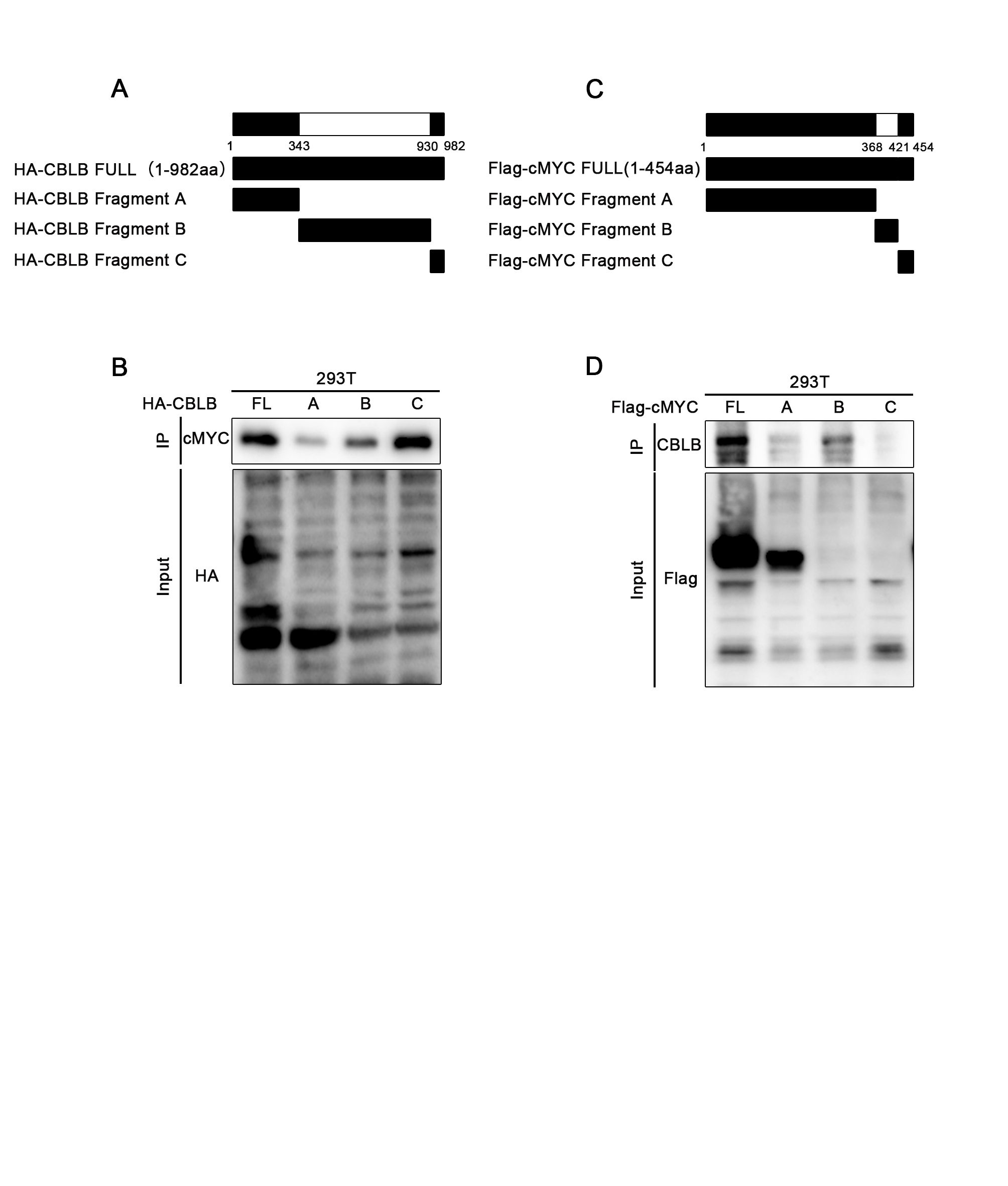

Supplement: Supplementary file 10 — Additional file 10: Figure. S10. MiR-223-3p targets E3 ligase CBLB to regulate ubiquitination of cMYC. (A-B). Immunoprecipitation of CBLB constructs and cMYC in 293T cells. (C-D). Immunoprecipitation of cMYC constructs and CBLB in 293T cells. [file 13046_2023_2921_MOESM10_ESM.png]

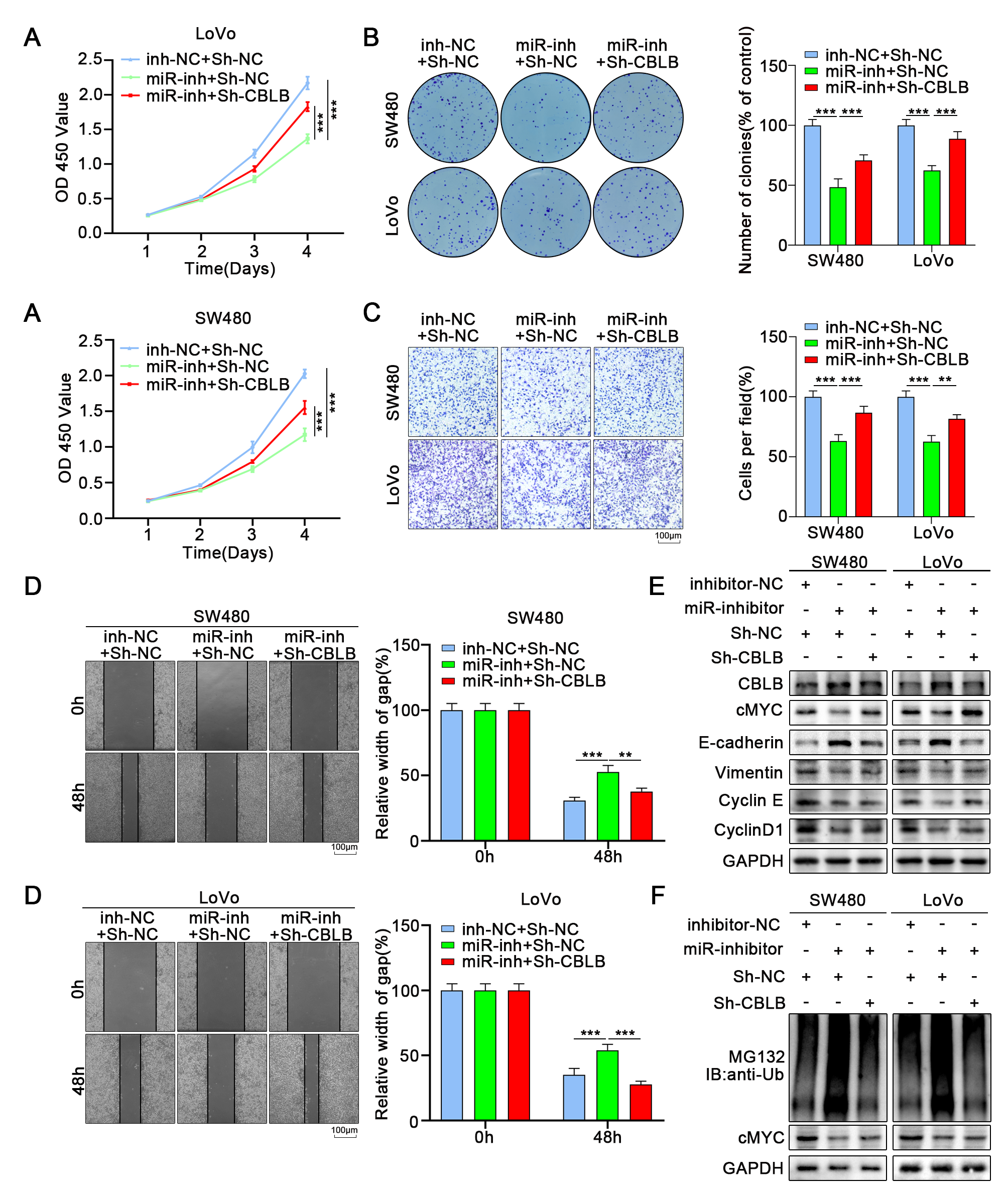

Supplement: Supplementary file 11 — Additional file 11: Figure. S11. GABA promotes proliferation and migration of CRC via miR-223-3p/CBLB/cMYC axis. A. CCK8 assays showed that CBLB knockdown reversed the effect of inhibitor of miR-223-3p on the inhibition of proliferation in colon cancer cells. B. The colony formation assay indicated that CBLB knockdown reversed the effect of inhibitor of miR-223-3p on the inhibition of proliferation in colon cancer cells. C. The transwell assays showed that CBLB knockdown reversed the effect of inhibitor of miR-223-3p on the inhibition of proliferation in colon cancer cells. D. The wound healing assays showed that CBLB knockdown reversed the effect of inhibitor of miR-223-3p on the inhibition of proliferation in colon cancer cells. E. Western blot indicated that CBLB knockdown reversed the effect of inhibitor of miR-223-3p on the inhibition of proliferation in colon cancer cells. F. Ubiqutin assays indicated that CBLB knockdown blocked the altered ubiquitination of cMYC in colon cancer induced by inhibitor of miR-223-3p. All data were revealed as mean ± standard deviation (SD) for no less than three independent experiments. Significant P values showed as ***P<0.001.**P <0.01. [file 13046_2023_2921_MOESM11_ESM.png]
